# Targeting Pin1 to overcome immunosuppressive tumor microenvironment in MSS colorectal cancer

**DOI:** 10.3389/fimmu.2025.1677029

**Published:** 2025-10-31

**Authors:** Jian Wang, Shuxin Tang, Jinhua Fan, Huihui Xiao, Shihua Wang, Jianqin Xiang, Zhili Liu, Hongying Liu, Zhigang Pei, Dequan Jiang, Leiyuan Shuai, Han Liu, Jian Ye, Jianli Xu, Chengyuan Qian, Siqian Cui, Debing Xiang, Chunrong Wu

**Affiliations:** ^1^ Department of Oncology, Chongqing University Jiangjin Hospital, Chongqing, China; ^2^ Department of Oncology, Jiangjin Central Hospital of Chongqing, Chongqing, China; ^3^ Department of Neonatology, The Sixth Affiliated Hospital, Sun Yat-sen University, Guangzhou, Guangdong, China; ^4^ Central Laboratory, Chongqing University Fuling Hospital, Chongqing University, Chongqing, China; ^5^ The First Medicine College, Chongqing Medical University, Chongqing, China; ^6^ Department of Cardiothoracic Surgery, Southwest Hospital Third Military Medical University, Chongqing, China; ^7^ Department of Pathology, Chongqing University Jiangjin Hospital, Chongqing, China; ^8^ Department of Gastrointestinal Surgery, Chongqing University Jiangjin Hospital, Chongqing, China; ^9^ Department of Anus and Intestine Surgery, Chongqing University Jiangjin Hospital, Chongqing, China; ^10^ Department of Ophthalmology, Chongqing University Jiangjin Hospital, Chongqing, China; ^11^ Department of Anesthesiology, Chongqing University Jiangjin Hospital, Chongqing, China; ^12^ Department of Oncology, Daping Hospital, Army Medical University, Chongqing, China; ^13^ College of Chemistry and Chemical Engineering, Chongqing University, Chongqing, China

**Keywords:** Pin1, MSS colorectal cancer (CRC), immunosuppressive tumor microenvironment (TME), immunotherapy resistance, NF-κB-CCL3-CCR5 axis

## Abstract

**Background:**

Management of the immunosuppressive tumor microenvironment (TME) is crucial for microsatellite stability (MSS) colorectal cancer (CRC), which responds poorly to immunotherapy. PIN1, a peptidyl-prolyl cis-trans isomerase that is overexpressed in human malignancies, regulates TME immunosuppression. However, its role in MSS CRC remains insufficiently explored.

**Methods:**

We divided 411 CRC patients from the TCGA-COAD database into MSS or microsatellite instability-high (MSI-H) groups and analyzed their gene expression profiles. Using smoothed t-statistic SVM, weighted correlation network analysis, and sample clustering, we identified PIN1 as a key biomarker. We assessed Pin1 expression in CRC cell lines and tissues, and conducted functional assays, *in vitro* co-cultures, and *in vivo* studies (using a Pin1 inhibitor and anti-PD-1 in CT26 subcutaneous tumor and liver metastasis mouse models) to evaluate its effects and mechanisms.

**Results:**

PIN1 was overexpressed in MSS CRC and negatively correlated with CD4+ T and CD8+ T cell infiltration. Knockdown of PIN1 in MSS CRC cells significantly reduced cell proliferation (assessed by CCK-8 assay), impaired migratory capacity (assessed via wound-healing assay), and increased the apoptotic rate (detected by flow cytometry). In CT26 mouse models, combining Pin1 inhibition with PD-1 blockade enhanced immunotherapy efficacy by reducing Treg infiltration, suppressing cancer-associated fibroblast (CAF) activity, and promoting CD8+ T cell recruitment. Mechanistically, PIN1 activated the NF-κB pathway and modulated CCL3-CCR5 signaling, which are critical for Treg migration and CAF activation.

**Conclusion:**

Our findings suggest that Pin1 reshapes the immunosuppressive TME in MSS CRC through the NF-κB-CCL3-CCR5 axis, driving CRC progression and immunotherapy resistance. This pathway presents a potential target for overcoming immunotherapy resistance in MSS CRC.

## 1 Introduction

Colorectal cancer (CRC), a globally prevalent malignant neoplasm, holds the third position in terms of incidence and mortality rates among all cancers, posing a significant health challenge worldwide (1). For patients with advanced or metastatic CRC (mCRC), chemotherapy and targeted therapies targeting vascular endothelial growth factor (VEGF) or epidermal growth factor receptors (EGFRs) are standard treatments. Despite these efforts, the prognosis for these patients is poor, with a 5-year survival rate of approximately 14% (2). The advent of immune checkpoint blockade (ICB) has transformed cancer therapeutics with the introduction of inhibitors of programmed cell death 1 (PD-1), programmed cell death ligand 1 (PD-L1), and cytotoxic T-lymphocyte antigen 4 (CTLA-4). In 2017, the U.S. Food and Drug Administration (FDA) approved the PD-1 antibodies pembrolizumab and nivolumab for the second-line treatment of CRC patients with deficient DNA mismatch repair/microsatellite instability-high (dMMR/MSI-H) (3). This was followed by the FDA’s approval of pembrolizumab in June 2020 for the first-line treatment of patients with unresectable or metastatic CRC with MSI-H. However, MSI-H is present in only 15% of CRC cases, whereas proficient mismatch repair and microsatellite stabilization (pMMR/MSS) is present in approximately 85% of CRC cases (4). The use of immunotherapy in CRC remains challenging, especially in patients without MSI-H. Therefore, strategies to improve efficacy and discover new biomarkers are the current focus of research.

The mechanisms underlying immunotherapy resistance in MSS CRC remain unclear. It has been hypothesized that a scarcity of immune cell infiltration and reduced tumor mutational burden (TMB) might be contributing factors (5). A higher density of CD3+ lymphocytes and CD8+ T cells has been linked to a decreased risk of recurrence, enhanced disease-free survival, and improved overall survival (6). Studies have indicated that a higher count of tumor-infiltrating lymphocytes correlates with better clinical prognosis (7). The tumor microenvironment (TME) composition and distribution of immune cell populations in MSI-H and MSS tumors are significantly different, which contributes to different treatment response rates and clinical prognoses (8). Tumors with MSI-H show greater immune cell infiltration, increased expression of genes associated with immunity, and higher immunogenicity than MSS tumors. They may also feature an inflammatory TME and show heightened sensitivity to immune checkpoint inhibitors (ICIs) (9). As a result, MSI-H tumors tend to respond better to treatment and have more favorable prognoses. In contrast, MSS tumors exhibit low mutational load, low immune cell recruitment and infiltration, low neoantigen burden, and poor immune response (10).

Therefore, a variety of novel combination therapies for MSS CRC are currently under investigation. Wang et al. conducted a randomized phase 2 trial, and concluded that the synergistic use of PD-1, HDAC, and VEGF inhibitors could be an effective treatment strategy for advanced MSS CRC patients (11). In another study, Fakih et al. initiated a phase 1 nonrandomized clinical trial to evaluate the combination of regorafenib, ipilimumab, and nivolumab (RIN) in patients with MSS CRC who had experienced disease progression after prior chemotherapy. The objective of this trial was to establish the recommended phase 2 dose for this combination (RIN) and to evaluate its efficacy in a larger group of MSS mCRC patients. The findings indicated significant clinical benefits for patients with advanced MSS CRC without liver metastases; however, these results require validation in future randomized clinical trials (12). Additionally, Fang et al. discovered that the combination of sintilimab with bevacizumab, oxaliplatin, and capecitabine demonstrated potential antitumor effects and a favorable safety profile as a first-line treatment for RAS-mutant, MSS, and unresectable mCRC (13). Hence, in-depth exploration of the molecular mechanisms that ameliorate immune resistance in MSS CRC is of great clinical value.

In this study, we analyzed gene expression profiles and corresponding clinical data from patients with CRC retrieved from The Cancer Genome Atlas (TCGA) database. PIN1 emerged as a significant candidate for further investigation. Using human tumor tissue samples, MSS CRC cells, and animal models, we systematically evaluated the role of Pin1 as a mediator of CRC immunotherapy resistance, with a specific focus on its ability to regulate the TME. Together, these results will provide foundation for novel immunotherapeutic approaches in MSS CRC, particularly when used in conjunction with ICB.

## 2 Methods

### 2.1 Patient samples and initial data processing

We obtained gene expression data of 411 CRC cases (including MSI-H and MSS) from TCGA-COAD. MSI-L was integrated into the MSS group due to its overlapping immunosuppressive TME features and similar immunotherapy response patterns with MSS. Single-cell RNA sequencing (scRNA-seq) data were retrieved from the public dataset (GSE178341) in the Gene Expression Omnibus (GEO). All data processing and initial analyses, including quality control, normalization, and preliminary differential expression assessment, were performed using R (version 4.3.2) unless stated otherwise.

### 2.2 Immune-related analysis

The relationships between PIN1 expression levels and the presence of CD8+ T and CD4+ T cells were quantified using the cor function from the R package psych (version 2.4.3), which calculates the Pearson correlation coefficients. Multiple testing correction was applied to the resulting P values using the false discovery rate (FDR) method. Scatter plots illustrating these correlations, complete with linear trend lines, were generated using the ggplot2 R package (version 3.5.0).

### 2.3 Network smoothed t-statistic SVM

We used the Network smoothed t-statistic Support Vector Machine (stSVM), a machine learning algorithm integrating protein-protein interaction (PPI) network information with gene expression data to enhance feature gene selection accuracy, to identify genes associated with microsatellite stability status (MSS vs MSI-H). The stSVM algorithm was implemented using the cv.stsvm function in the R package netClass (version 1.2.1). The performance and generalization capacity of the model were evaluated by repeating 10-fold cross-validation times. Before performing stSVM analysis, we normalized the gene expression data. An adjacency matrix of gene interactions containing prior information on the protein-protein interaction (PPI) network was then constructed. The adjacency and gene expression matrix were integrated into a classifier; feature selection was performed based on sample groups (MSI-H vs. MSS), and genes associated with microsatellite stability status were identified and used to train the classifier. The R package ComplexHeatmap (version 2.18.0) was used to generate complex heat maps of the selected genes.

### 2.4 Weighted gene co-expression network analysis

We employed the R package WGCNA (version 1.72.5) to conduct co-expression network analysis, aiming to identify the most significant gene modules and key hub genes. Identification of hub genes is crucial for deciphering the core molecular mechanisms underlying phenotypic differences between MSS and MSI-H CRC, as hub genes are highly connected within co-expression networks and are more likely to play pivotal roles in regulating biological processes. The initial step involved applying the hclust function for hierarchical clustering of gene expression profiles, with the resulting dendrogram visualized as a sample clustering tree. Following this, the pickSoftThreshold function was utilized to identify the optimal soft threshold to approximate a scale-free network topology, which is a prerequisite for constructing the gene co-expression network. The blockwiseModules function was used to facilitate the formation of gene co-expression modules based on the selected threshold. Through cluster analysis, Genes were clustered into distinct modules, each assigned a unique color. The plotDendroAndColors function was used to combine the sample clustering tree and module color information for visualization. We calculated the correlation between module eigengenes (MEs) and clinical phenotypes, namely MSI-H and MSS, and utilized the labeledHeatmap function to present a heatmap depicting these correlations. A module of particular interest was selected, and constituent genes were extracted to form a subnetwork. The R package igraph (version 2.0.3) was then used to construct a minimum spanning tree for this subnetwork, which was subsequently visualized using the Cytoscape software (version 3.10.2). Finally, box plots for hub genes were generated using the ggplot2 R package (version 3.5.0) to graphically represent their distribution and significance.

### 2.5 Enrichment analysis

Using the R package clusterProfiler (version 4.10.1), we conducted GO and KEGG enrichment analyses on the genes identified from the stSVM algorithm and module genes that were most closely related to the MSI-H and MSS subgroups, as identified by WGCNA. The gene IDs were processed and converted using the bitr function from the R package stringr (version 1.5.1), which facilitated the conversion of gene SYMBOL to ENTREZ ID. For GO analysis, we employed org.Hs.eg.db, a curated human gene database, and applied the enrichGO function to scrutinize the genes in terms of biological process (BP), cellular component (CC), and molecular function (MF). KEGG analysis was executed using the enrichKEGG function, leveraging a species-specific library (has) for enrichment analysis. Finally, the outcomes of both GO and KEGG analyses were graphically represented using the dotplot function from the enrichplot R package (version 1.22.0), providing a clear and comprehensive visualization of the enrichment results.

### 2.6 Single-cell RNA sequencing data analysis

The scRNA-seq data were pre-processed using Seurat v4.1.1. We applied the Uniform Manifold Approximation and Projection (UMAP) algorithm for dimensionality reduction and annotated cell types based on markers referenced in the original publication (14). Seven major cell subpopulations were identified: Epithelial cells (Epi), Myeloid, B, Plasma, Stromal, Mast, and T/NK/ILC subpopulations. We used the ggplot2 package to create a stacked bar chart illustrating the relative proportions of different cell subpopulations within each sample. Median expression values, normalized using Seurat’s ScaleData function, were used to segregate cells into high and low PIN1 expression groups. Within these groups, we further examined the distribution of CD4 +T cells, CD8+ T cells, Treg cells, M2 macrophages, and fibroblasts to elucidate the immunological landscape associated with varying levels of PIN1 expression.

### 2.7 Cell culture and cell transfection

Three human MSS CRC cell lines (HT29, SW480, and SW620) and two MSI-H CRC cell lines (HCT116 and HCT15) were cultured in DMEM (Gibco, USA) supplemented with 10% FBS (VivaCell, China) and 1% penicillin-streptomycin (Gibco, USA). The murine MSS CRC cell line (CT26) was cultured in RPMI 1640 (Gibco, USA) and the MSI-H CRC cell line (MC38) was cultured in DMEM (Gibco, USA) supplemented with 10% FBS (VivaCell, China) and 1% penicillin-streptomycin (Gibco, USA). All cells were obtained from Procell (Wuhan, China). CAFs were obtained from iCell (Shanghai, China) and cultured in their recommended Primary Fibroblast Culture System (PriMed-iCell-003, Shanghai, China). Two specific siRNAs targeting PIN1 and a scrambled siRNA were synthesized by HippoBio (Huzhou, China). SW480 and HT29 cells were transfected using the Lipofectamine 3000 kit (Invitrogen, USA), according to the manufacturer’s protocol. Changes in PIN1 mRNA expression were assessed by qRT-PCR at 24 h post-transfection, and changes in Pin1 protein levels were analyzed by western blotting 48 h post-transfection. The verified siRNAs for human PIN1 (PIN1-1: 5’-CGGCTACATCCAGAAGATCAA-3’; PIN1-2: 5’-CAGGCCGAGTGTACTACTT CA-3’) were synthesized as previously described (15). A scrambled siRNA: 5’-UUCUCCGAACGUGUCACGUT T-3’, served as a negative control.

### 2.8 qRT-PCR, CCK8, wound-healing, apoptosis, ELISA and co-immunoprecipitation assays

qRT-PCR, CCK8, wound healing, and apoptosis assays were performed according to established protocols (16). The primers used are listed in **Supplementary Table S1**. The concentrations of CCL3 in culture media were evaluated using Human CCL3 (MIP-1a) ELISA Kit (liankebio, cat.no. EK161-AW1), according to the manufacturer’s instructions.

In the Co-IP assay, cellular lysis was achieved using IP lysis buffer (Beyotime, China; cat.no. P0013J), to which phosphatase and protease inhibitors were freshly added. After centrifugation at 12,000 × g for 15 min, a portion of the supernatant was reserved as the input, whereas the rest was incubated with 2 µg of anti-Pin1 antibody (Santa Cruz, USA; sc-46660) for 12 h at 4 °C. The lysate-antibody mixtures were further incubated with Protein A/G agarose beads (Beyotime, cat.no. P2055) for 2 h at 4 °C, after which the beads were washed five times with the same lysis buffer. The immunoprecipitates, collected after brief centrifugation, were then resuspended in 2 × SDS loading buffer (Beyotime, China; cat.no. P0015B), heated at 95 °C for 10 min, and finally analyzed by western blotting.

### 2.9 Western blotting, immunohistochemistry, and multiple immunohistochemistry assays

To determine the expression of relevant indicators and their positions within the CRC, samples were collected from CRC patients undergoing surgery at Chongqing University Jiangjin Hospital (Chongqing, China). Participants were selected based on specific criteria: inclusion required a confirmed primary CRC diagnosis, genetic testing for MSS or MSI-H subtypes, and no history of other cancers; exclusion involved severe organ dysfunction, active infections, immune diseases, or prior treatments such as chemotherapy or radiation. Patient clinical and pathologic characteristics are shown in **Supplementary Table S2**. All patients gave written consent, and the study received ethical approval from Jiangjin Central Hospital’s Ethics Committee (KY20240812-003).

Western blotting was performed according to previously established methods (16). For IHC assays, sections of CRC tissue embedded in paraffin (4 μm thick) were deparaffinized and rehydrated, followed by antigen retrieval. After non-specific antigen blocking, the sections were incubated with primary antibodies at 4 °C overnight, rinsed, and incubated with secondary antibodies for 1 h at 37 °C. The reaction was developed using diaminobenzidine (DAB), and the sections were counterstained with hematoxylin. Positive and negative controls were included in the IHC analysis for each antibody. As described in our previous study (16), IHC scoring was conducted by assessing the percentage of stained cells (1 = <25%, 2 = 25-50%, 3 = 51-75%, and 4 = >75%) and multiplying this percentage score by the staining intensity (0, 1, 2, or 3) to yield a final possible score of 0–12. Scores < 4 were considered to indicate low levels of Pin1 expression, while all other scores were indicative of high expression. Two experienced pathologists blinded to patient characteristics independently scored all samples.

In the mIHC assays, both human CRC and murine tumor tissue sections were subjected to antigen retrieval by boiling in a solution of 10 mM sodium citrate buffer at pH 6.0, following deparaffinization. The sections were then permeabilized with PBS containing 0.5% Triton X-100 and blocked with PBS supplemented with 5% goat serum for 30 min at room temperature. The primary antibodies were diluted in PBS with 1% goat serum, and the slides were incubated at 4 °C overnight. HRP-conjugated polymer anti-rabbit antibody was used as the secondary antibody. Finally, a fluorophore-conjugated tyramide signal amplification buffer (AFIHC034, AiFang Chemical, China) was applied and DAPI was used as a nuclear counterstain. The primary antibodies used are listed in **Supplementary Table S3**. All procedures involving human participants were performed in accordance with the ethical standards of the institutional research committee and the Helsinki Declaration.

### 2.10 Immunofluorescence analysis

Cell cultures were initiated by seeding onto chamber slides, followed by fixation using 4% paraformaldehyde solution for 20 min. Subsequently, the slides underwent three cycles of washing, each with PBS for 5 minutes. To enhance membrane permeability, cells were treated with 0.1% Triton X-100, followed by blocking with 3% BSA in PBS for 30 min at room temperature. After a series of PBS washes, the slides were incubated with primary antibodies overnight at 4 °C. The following day, the slides were treated with either Dylight 488-conjugated goat anti-rabbit IgG (Earthox, USA) or Dylight 649-conjugated goat anti-mouse IgG (Earthox, USA) for 1 h in a dark chamber. Finally, the slides were stained with a DAPI solution containing anti-fluorescence quenching agents prior to examination under a fluorescence microscope. The details of the primary antibodies used in this process are listed in **Supplementary Table S3**.

### 2.11 Animal model

For Pin1i treatment in mice model, twenty male BALB/c mice (6–8 weeks old) were procured from Huachuang Sino (Jiangsu, China). CT26 cells (1 × 10^6^) were subcutaneously injected into mice. Once the tumor volumes reached approximately 125 mm^3^, the mice were randomized into the following groups: N.S. + IgG, N.S. + anti-PD1, Pin1i + IgG, and Pin1i + anti-PD-1 (n = 5/group). The mice were treated daily with sulfopin (40 mg/kg, i.p., MCE, cat.no. HY-139361), N.S (sulfopin diluted solution; 45% saline, 40% PEG300, 10% DMSO, and 5% Tween 80), and anti-PD-1 every 3 days (200 μg, i.p., Leinco, cat.no. BE0146), or IgG isotype control (Leinco, cat.no. BE0090). Tumor volume was measured every three days and calculated using the following formula: V = a × b × b/2. CRC liver metastasis model was established via intrasplenic injection of CT26 cells (2 × 10^5^ cells). Starting from day 7 post-injection, the Pin1 inhibitor was administered daily, while the PD-1 antibody was injected every 3 days. On day 14 after the initiation of treatment, mice were euthanized, and their livers were harvested for histological evaluation. Investigators performing measurements/drug administration were blinded to group allocation. All experimental procedures and protocols involving the use of mice were reviewed and approved by the Experimental Animal Welfare Ethics Committee of Chongqing University Cancer Hospital (No. CQCH-LAE-20231020016).

### 2.12 Flow cytometric analysis

To explore immune cell infiltration in tumors, mouse tumor tissues were finely sliced into 0.5-1.0 mm fragments and dissolved using 0.5 mg/ml collagenase IV (Invitrogen, cat.no. 17104019) and 0.1 mg/ml DNase I (Sigma, cat.no. 10104159001) for 40 min at 37 °C, according to the manufacturer’s instructions. The suspensions were passed through 70 μm cell strainers to filter out the debris. Subsequently, the cells were incubated with anti-mouse CD16/32 (BioLegend; cat.no.156604) to block non-specific binding and then stained using the Zombie Green™ Fixable Viability Kit (Biolegend; cat.no.423101) and specific antibodies in PBS containing 5% FBS. The stained cells were measured using CytoflexLX flow cytometer (Beckman Coulter, USA), and data were analyzed using FlowJo V10. Specific antibodies used in this process were procured from Invitrogen, and a detailed list is presented in **Supplementary Table S2**. Gating strategies are presented in **Supplementary Figure S1**.

### 2.13 Co-culture of peripheral blood mononuclear cells and CRC cells and chemotactic assays

For the indirect co-culture model, PBMCs were isolated from six healthy volunteers (three males and three females) by density gradient centrifugation using Ficoll (Solaibao, China; catalog no. P8610), with approval from the Ethics Committee of Jiangjin Central Hospital of Chongqing (KY20240812-003). The cells were then activated for 72 h with IL-2 (10 ng/ml, Peprotech, USA; cat.no.200-02) and CD3/CD28 magnetic beads (T&L Biotechnology, China; cat.no.SMP-SHC-002-F-003) in RPMI-1640 medium (Gibco, USA) supplemented with 10% FBS (VivaCell, China) and 1% penicillin-streptomycin (Gibco, USA). CRC cells (SW480 or HT29) with or without Pin1 interference were co-cultured indirectly with activated PBMCs in 24­well transwell co-culture plates (0.4 μm polyester film) for 48 h. PBMCs were plated in the upper layer, and CRC cells were plated in the lower layer. The preparation of conditioned media from SW480 and HT29 cells and subsequent chemotactic assays were performed as previously described (17).

### 2.14 *In vitro* Treg cell migration assay

PBMCs were purified and activated overnight. After washing and removing the magnetic CD3/CD28 beads, the cells were resuspended in PBS and stained with CD4, CD8, CD25, and CD127 monoclonal antibodies for 30 min. Treg cells were defined as those expressing CD4 and CD25 while exhibiting negative or low levels of CD127 (CD4+ CD25+ CD127-/low) and were subsequently sorted using flow cytometry. Isolated Treg cells (1 × 10^5^) were then resuspended in 100 μl of serum-free medium and placed into the upper compartment of 5-μm pore size transwell inserts (Corning, USA). The inserts were transferred to 24-well plates filled with 600 μl of cell-conditioned medium and incubated for 4–5 h in a cell incubator. The number of cells that migrated to the lower chamber was quantified. Three independent experiments were performed for each assay.

### 2.15 Statistical analysis

All *in vitro* experiments were performed independently at least three times, with sample sizes determined by evaluating sample variability and means. In the *in vivo* studies, the animals were randomly assigned to groups, and the assessment of outcomes was not blinded. Statistical analysis was performed utilizing Prism 5 software (CA, USA). Data are expressed as mean ± standard deviation. The significance of differences was assessed using unpaired Student’s t-tests for two-group comparisons (e.g., differences in PIN1 expression between MSS and MSI-H), one-way ANOVA for multiple groups (e.g., four treatment groups in murine models), and Pearson’s chi-square tests for categorical variables (e.g., the relationship between PIN1 expression level and clinical stage). Pearson correlation coefficients were used to analyze linear relationships between gene expression levels.

## 3 Results

### 3.1 Differential cellular composition and microenvironmental insights into MSS and MSI-H CRC

**Figure 1:**
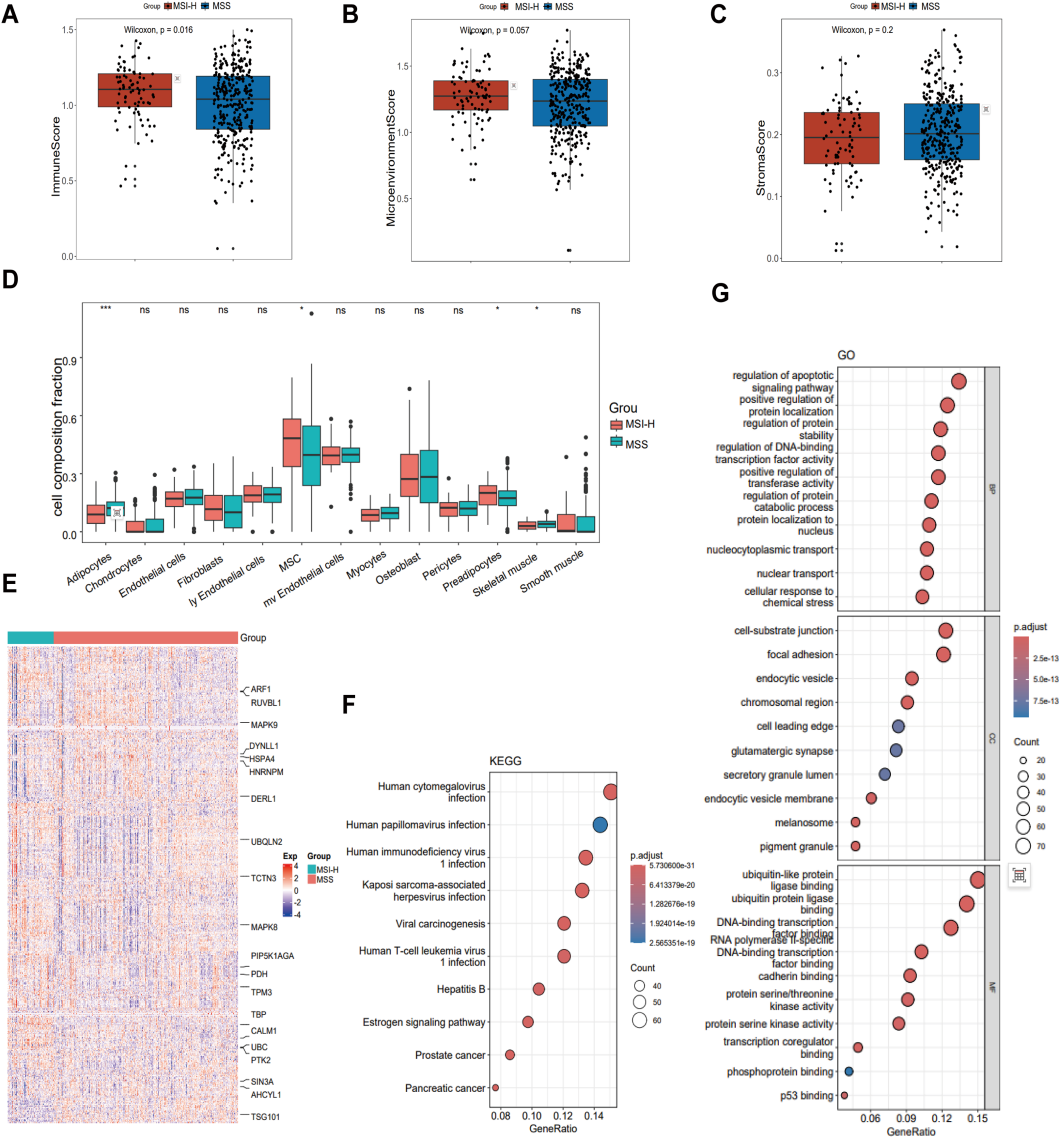
Comparison of the tumor microenvironment (TME) in MSS and MSI CRC. **(A-C)** The immune score **(A)** and microenvironment score **(B)** were higher in MSI-H CRC (P = 0.016 and P = 0.057, respectively), while the MSS group demonstrated a higher stroma score **(C)** than the MSI-H group (P = 0.2). **(D)** Comparison of cellular composition associated with tumor stroma in MSI-H and MSS CRC reveals significant differences in adipocytes, MSC, preadipocytes and Skeletal muscle. **(E)** Heatmap showing 530 MSS/MSI-related feature genes, exemplified by ARF1, RUVBL1, and MAPK9. **(F)** KEGG was performed on 530 feature genes. **(G)** GO was performed on 530 feature genes. *p < 0.05; ***p < 0.001.

In this study, we obtained gene expression profiles from 411 patients with CRC enrolled in TCGA-COAD dataset. To identify biomarkers associated with CRC, the patients were divided into two groups: MSS and MSI-H. To understand the cellular composition and TME of the MSS and MSI-H subgroups, xCell was used to infer and quantify 64 distinct cell types. In our analysis, the MSS and MSI-H tumor subgroups exhibited significant differences in immune scores, with MSI-H tumors having a higher score (*p* = 0.016) (**Figure 1A**). Consistent with this, a trend toward a higher microenvironment score was observed in the MSI-H group compared to the MSS group (*p* = 0.057) (**Figure 1B**), while the MSS group demonstrated a higher stroma score than the MSI-H group (*p* = 0.2) (**Figure 1C**). To further understand the cellular types associated with the tumor stroma, we extracted data from the xCell analysis of stroma-related cell types. The results revealed significant differences in adipocytes, MSCs, preadipocytes, and Skeletal muscle cells between the MSS and MSI-H tumor samples (**Figure 1D**).

We used the stSVM algorithm to enhance the identification of CRC-related biomarkers. We integrated the PPI network with the gene expression matrix, conducted feature training, and identified 530 grouping-related genes (**Figure 1E**). To uncover the key enriched pathways associated with the feature genes related to the stability of microsatellites in the tumor genome status, we used KEGG and GO analyses of the feature genes obtained from the stSVM algorithm. KEGG analysis results revealed that the feature genes associated with the MSS and MSI-H subgroups were primarily enriched in viral infections pathways (cytomegalovirus and papillomavirus) and cancer-related signaling pathways (including prostate cancer, pancreatic cancer, and viral carcinogenesis) (**Figure 1F**). GO analysis showed enrichment primarily in transcription factor activity, apoptosis signaling, protein localization and stability, and in key molecular mechanisms such as p53 binding (**Figure 1G**).

### 3.2 Identification of hub genes in MSS and MSI-H CRC through WGCNA

**Figure 2:**
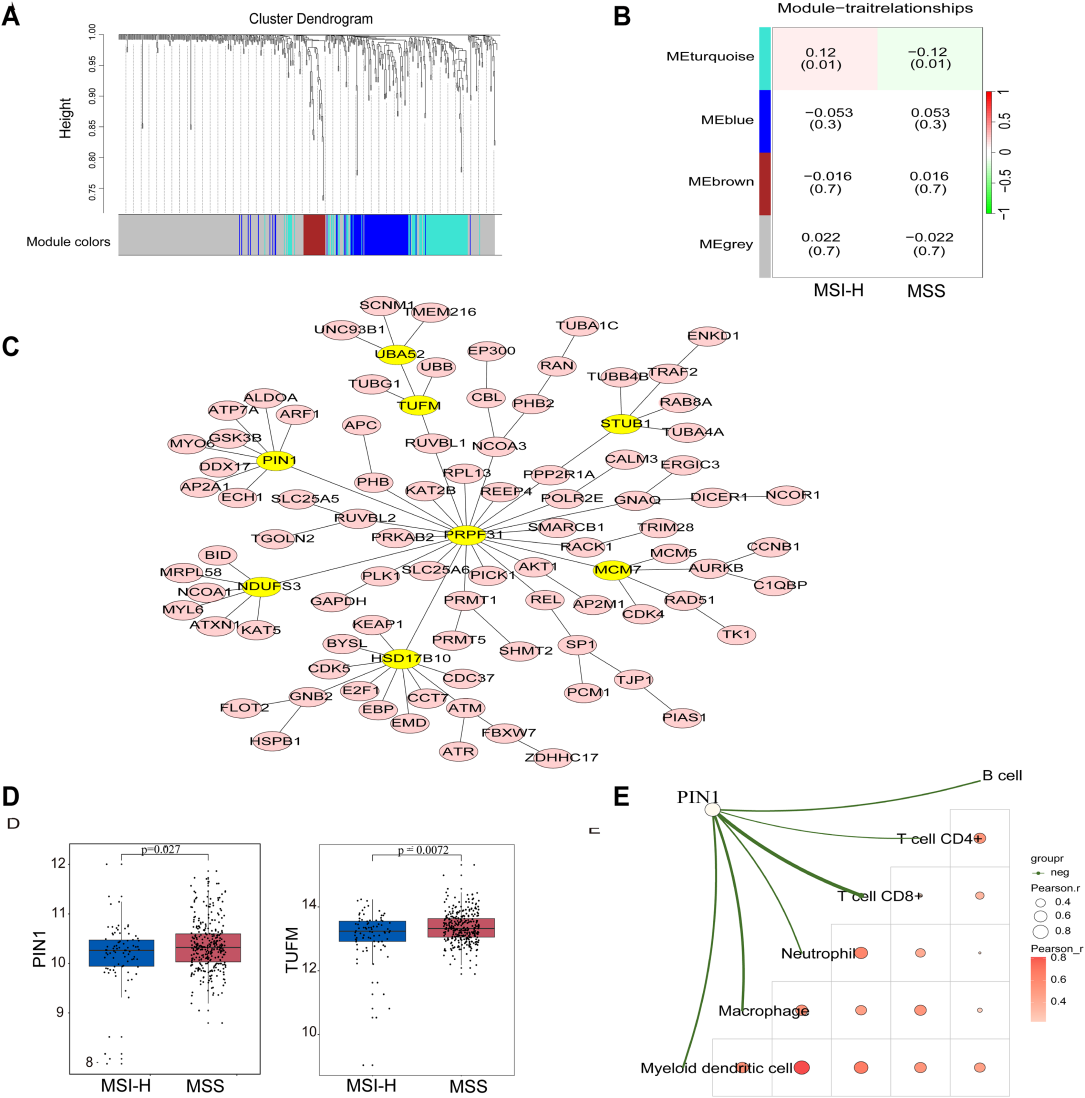
Identification of hub genes in MSS and MSI CRC through WGCNA analysis. **(A)** The Cluster dendrogram of WGCNA. **(B)** Analysis of correlations between the modules and MSS/MSI-H CRC samples; p-values are shown. **(C)** The minimum spanning tree was used to construct the co-expression network of the turquoise module genes, where yellow ovals indicate the hub genes. **(D)** Expression of PIN1 and TUFM in the MSS/MSI-H CRC samples. **(E)** The correlation analysis between PIN1 expression and CD8+T, CD4+T neutrophils, macrophages, and myeloid dendritic cells. The green line in the schematic represents a negative correlation between gene expression and immunity scores. A deeper red indicates a higher correlation, and a bigger circle signifies a stronger correlation.

To identify hub genes associated with MSS and MSI-H subgroups, we conducted WGCNA. We performed cluster analysis on the samples, visualized the relationship between phenotype and gene expression data, and reconstructed the sample clustering tree (**Figure 2A**). **Figure 2B** shows that among the four modules (turquoise, blue, brown, and gray), the turquoise module exhibited a significant correlation with the MSS and MSI-H tumor samples (cor = ± 0.12, *p* = 0.01) (**Figure 2B**). The final co-expression network of the turquoise module genes was constructed using the standard minimum spanning tree approach. PIN1, PRPF31, TUFM, UBA52, STUB1, NDUFS3, HSD17B10, and MCM7 were identified as hub genes in this network (**Figure 2C**). Both PIN1 and the TUFM were highly expressed in MSS CRC samples (**Figure 2D**). Immune score correlation analysis revealed a negative correlation between PIN1 expression and immune cells (CD8+T cells, CD4+T cells, neutrophils, macrophages, and myeloid dendritic cells) (**Figure 2E**). These results indicate that PIN1 regulates immune response to CRC.

### 3.3 Pin1 was highly expressed and promoted cell proliferation, migration, and inhibited apoptosis in human MSS CRC

**Figure 3:**
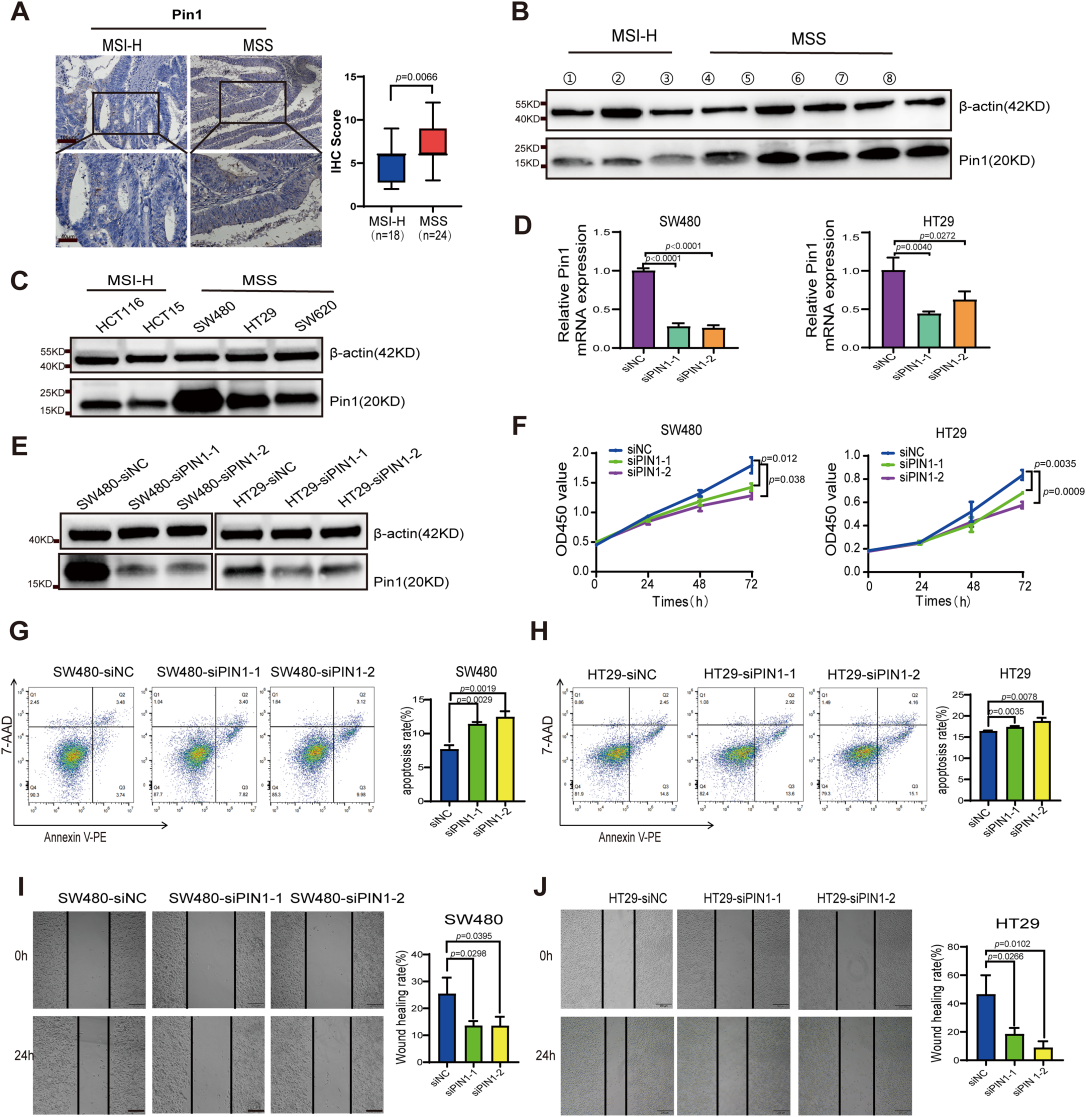
Analysis of Pin1 expression in CRC tumors and cell lines, and functional validation of Pin1 in MSS CRC cells. **(A)** Representative IHC staining of Pin1 and analysis of IHC score in MSI and MSS CRC tissues. **(B)** Pin1 protein levels in MSI and MSS CRC tissues were detected using western blot assay. **(C)** Pin1 protein levels in MSI-H (HCT116 and HCT15) and MSS (SW480, HT29, and SW620) CRC cell lines were detected using western blot assay. **(D, E)** qRT-PCR and western blot were performed to assess Pin1 knockdown. **(F)** The proliferation rates of Pin1-knockdown and control cells as tested using CCK-8 assay. **(G, H)** Assessment of apoptosis via flow cytometry. **(I, J)** The migratory capacity of Pin1-knockdown and control cells was evaluated via wound-healing assay.

PIN1 is recognized as a master regulator of malignant processes and is closely associated with tumor cell metabolic reprogramming, proliferation, migration, drug resistance, stem cell-like characteristics, and TME regulation (18–20). However, there is a lack of research regarding its involvement in CRC, particularly MSS CRC. Using IHC, we examined Pin1 expression in both MSS and MSI-H CRC tissues and observed elevated expression in MSS CRC samples (**Figure 3A**). WB was conducted to compare Pin1 expression across three MSI-H CRC and five MSS CRC tissue samples, revealing that Pin1 levels were higher in MSS CRC tissues than in MSI-H CRC tissues (**Figure 3B**, **Supplementary Figure S2**). Additional validation across multiple cell lines indicated that Pin1 protein levels were more abundant in MSS CRC cell lines (SW480, HT29, and SW620) than in MSI-H CRC cell lines (HCT116 and HCT15) (**Figure 3C**, **Supplementary Figure S2**). Mouse MSS CRC cells (CT26) also showed higher Pin1 expression levels than mouse MSI-H CRC cells (MC38) (**Supplementary Figures S2**, **S3**). Collectively, these results confirmed the elevated Pin1 expression in MSS CRC.

To assess the biological functions of Pin1 *in vitro*, MSS CRC cells with highest Pin1 expression (SW480 and HT29) were transfected with PIN1-targeting siRNA, with scrambled siRNA serving as the control. Pin1 expression was effectively reduced in SW480 and HT29 cells by siRNA (**Figures 3D, E**, **Supplementary Figure S2**). We evaluated the effect of Pin1 knockdown on CRC cell proliferation using CCK-8 assays, which showed a marked reduction in proliferation in SW480 and HT29 cells (**Figure 3F**). Flow cytometry analysis of apoptosis revealed that Pin1 downregulation significantly increased apoptosis in these cells (**Figures 3G, H**). Wound healing assays demonstrated that Pin1 knockdown impaired migration in SW480 and HT29 cells (**Figures 3I, J**). These results confirm that Pin1 promotes oncogenic phenotypes in MSS CRC cells.

### 3.4 Pin1 inhibitor reduced Treg infiltration and increased CD8+T cell infiltration

**Figure 4:**
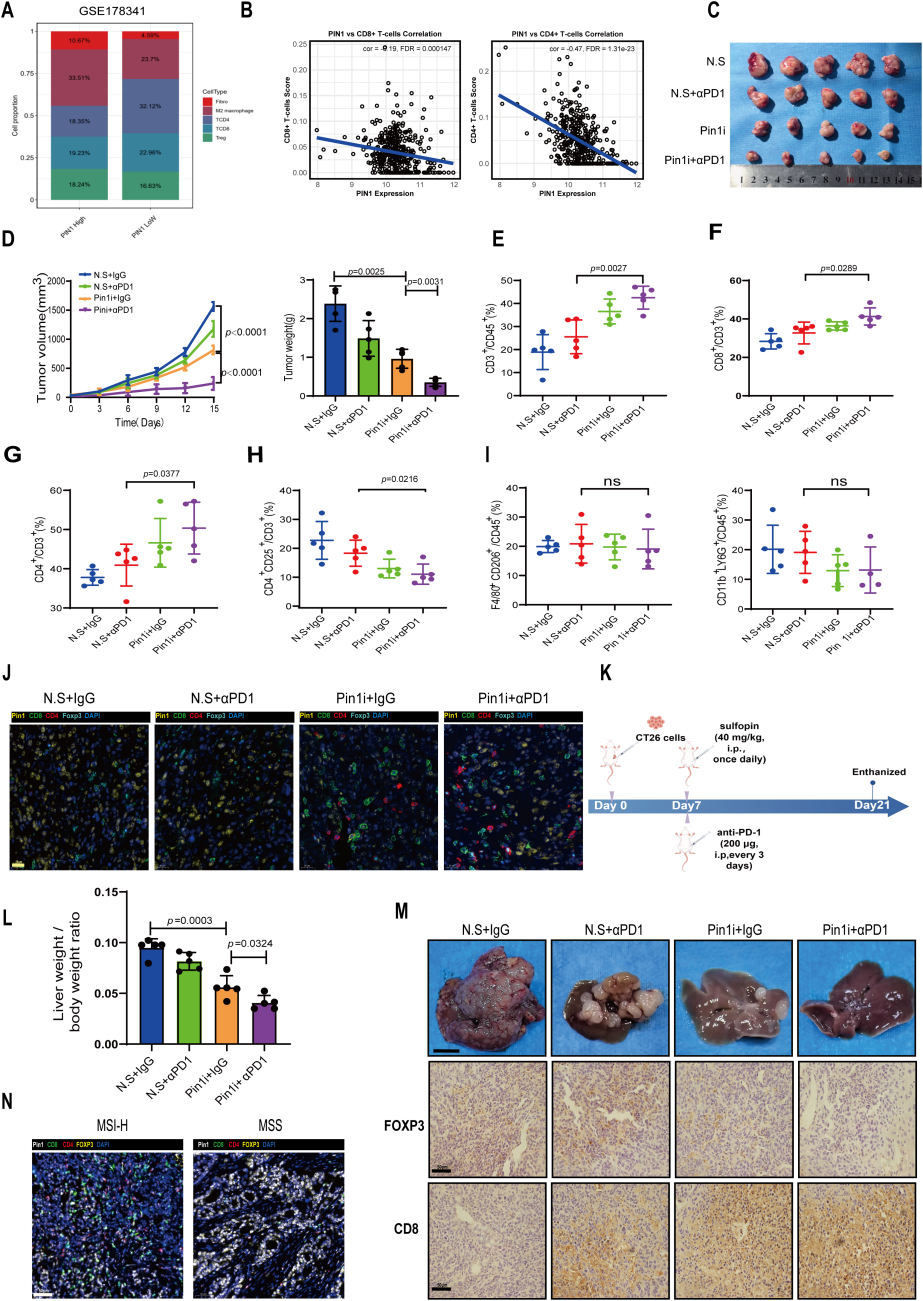
Pin1 inhibitor combined with ICB enhances antitumor immunity by blocking Treg recruitment and activating CD8+ T cells. **(A)** In the GSE178341 dataset, the MSS samples were divided into a high- and low- Pin1 expression group using the median Pin1 expression level, and the immune cell infiltration was compared between the two. **(B)** Analysis of the correlation between Pin1 and CD8/CD4+ T cells. **(C)** BALB/c mice were injected with CT26 cells subcutaneously. The mice were treated with N.S. (Sulfopin diluted solution; 45% saline, 40% PEG300, 10% DMSO, and 5% Tween 80), N.S. + αPD1, Pin1 inhibitor (Sulfopin, 40mg/kg), or Pin1 inhibitor + αPD1 (n = 5 each group). Tumors removed from the mice after sacrifice are shown. **(D)** Tumor volume, growth, and weight in each group. **(E)** The proportion of CD3+ T cells within the total CD45+ cell population in tumors is assessed using flow cytometry. **(F-H)** Flow cytometry assessing the proportion of CD8+ T cells, CD4+ T cells, and CD4+CD25+T cells in CD3+ cells in tumors. **(I)** Flow cytometry assessing the proportion of M2 macrophages and neutrophils in tumors. **(J)** Representative mIHC staining images of Pin1 and tumor-infiltrating immune cell populations (Foxp3+ Tregs, CD8+T cells, and CD4+T cells) in subcutaneous tumors from mice (n=5 per group). **(K)** Schematic diagram of intrasplenic injection mice model. **(L)** The ratio of liver/body weight of mice (n=5). **(M)** Representative images of indicated tumors. Scale bar: 1 cm. IHC analysis of FOXP3 and CD8 expression in hepatic metastasis mice tissue (n=5 per group). **(N)** Representative images of mIHC staining for Pin1 and immune cell populations, including Foxp3+ Tregs, CD8+ T cells, and CD4+ T cells (MSI-H, n=18; MSS, n=24).

To investigate PIN1 expression in scRNA-seq data, we analyzed the GSE178341 dataset comprising 371,223 tumor and adjacent normal cells from treatment-naïve CRC patients classified as MSS or MSI-H. We employed UMAP for dimensionality reduction and cell clustering, identifying distinct subpopulations based on gene expression profiles (**Supplementary Figures S4A, B**). Variation in cell type proportions across samples reflects CRC TME heterogeneity (**Supplementary Figure S4C**). The fluctuation in epithelial cell proportions may be associated with the tumor proliferative or metastatic potential. Using the normalized median of PIN1 expression level as a threshold, we divided the MSS samples into high- and low- PIN1 expression groups. Analysis revealed that the high-PIN1 expression group exhibited a lower proportion of CD4+ T cells and CD8+ T cells but a higher proportion of Treg cells, M2 macrophages, and fibroblasts (**Figure 4A**). PIN1 expression was negatively correlated with infiltration of CD8+ T and CD4+ T cells (**Figure 4B**).

We subcutaneously inoculated CT26 cells into mice and injected Pin1 inhibitor or αPD1/Pin1 inhibitor combination therapy into tumor-bearing mice (**Figure 4C**). Our findings indicate that Pin1 inhibitor monotherapy significantly reduced tumor volume and weight, while combination therapy produced stronger inhibition (**Figure 4D**). Flow cytometry analysis of tumor-infiltrating immune cells showed that Pin1 inhibitor monotherapy increased infiltration of CD3+ lymphocytes, CD8+ T cells, and CD4+ T cells. Combination therapy further enhanced infiltration of these cells (**Figures 4E–G**). Treg infiltration decreased after Pin1 inhibitor treatment and reached even lower levels with combination therapy (**Figure 4H**). Neither monotherapy nor combination therapy altered M2 macrophage or neutrophil infiltration (**Figure 4I**). Correspondingly, mIHC assay revealed fewer Foxp3+ Tregs and more CD8+ T and CD4+ T cells accumulated in Pin1 inhibitor-treated tumors, with amplified effects following combination therapy (**Figure 4J**).

We injected CT26 cells into the spleens of Balb/C mice to establish a hepatic metastasis model. Starting from day 7 post tumor inoculation, the mice received daily injections of the Pin1 inhibitor and injections of the anti-PD-1 antibody every 3 days. On day 14 after the initiation of treatment, hepatic metastatic burden was assessed (**Figure 4K**). The results demonstrated that the combined therapy of Pin1 inhibitor and anti-PD-1 antibody significantly suppressed tumor progression (**Figure 4L**). Consistent with our findings in mouse subcutaneous tumors, this combined therapy showed a significant decrease in Foxp3+ Tregs and a marked increase in CD8+ T cell infiltration in hepatic metastasis mice tissues (**Figure 4M**). In human MSS/MSI-H CRC specimens, higher Pin1 expression in MSS CRC correlated with increased Foxp3+ Treg infiltration and decreased CD8+ T cell infiltration (**Figure 4N**). These findings demonstrate that Pin1 inhibitor synergizes with ICB to enhance antitumor immunity in MSS CRC by suppressing Treg recruitment and activating CD8+ T cells in the TME.

### 3.5 Pin1 affected the chemotaxis of Tregs by regulating the CCL3-CCR5 pathway

**Figure 5:**
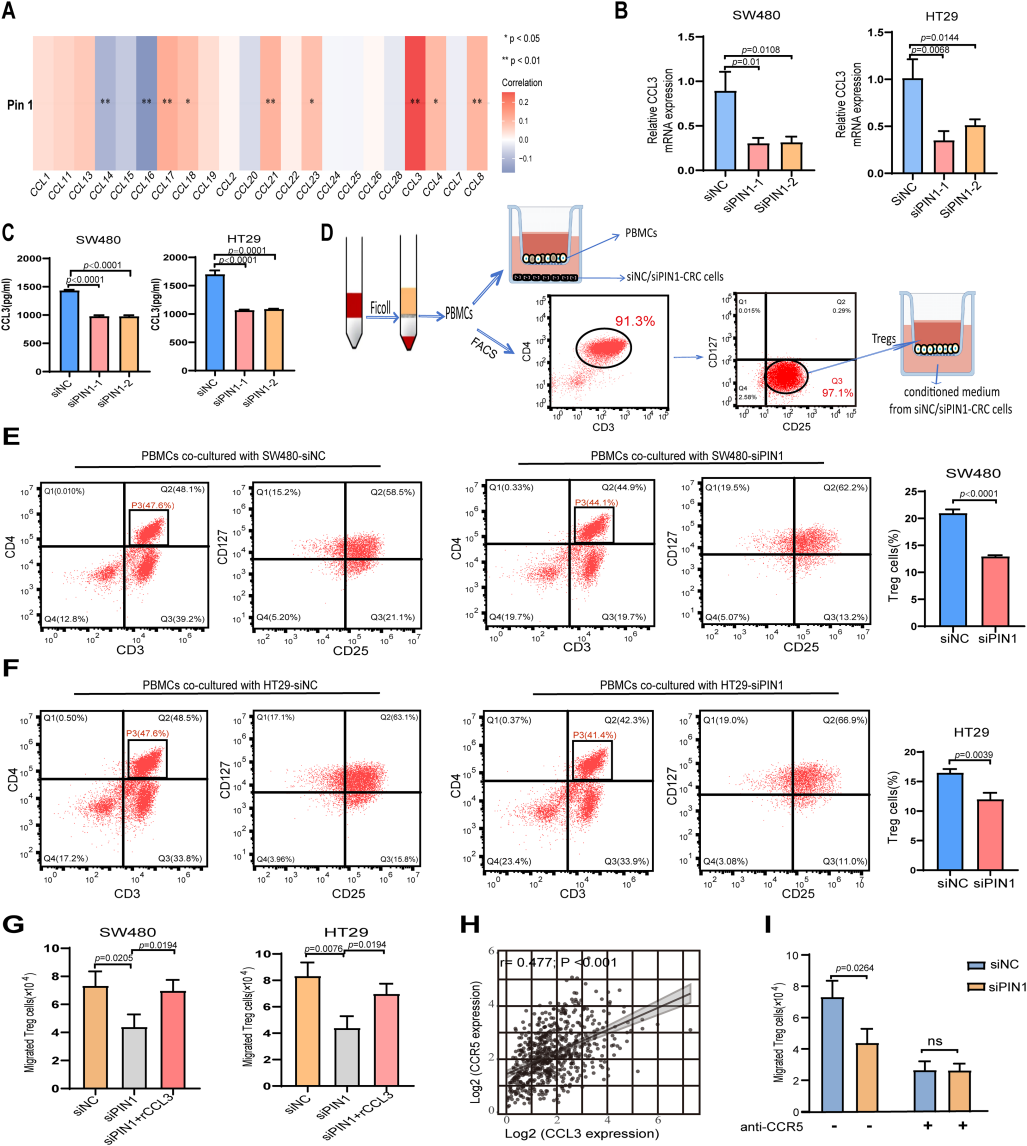
Pin1 promotes Tregs chemotactic migration through the CCL3-CCR5 axis. **(A)** Correlation analysis of Pin1 with CCL chemokines expression. **(B, C)** qRT-PCR and ELISA assessing CCL3 expression in SW480 and HT29 cells after Pin1 knockdown. **(D)** Schematic representation of PBMCs isolated from human peripheral blood; Treg cells sorted by flow cytometry, these populations co-cultured with siNC- or siPin1-CRC cells, respectively. **(E, F)** Level of Treg markers (CD25^+^CD127^-^) on PBMCs in the co-culture system detected by flow cytometry. **(G)** Effect of supernatants from Pin1-knockdown cells treated with recombinant CCL3 protein on Treg chemotactic capacity. **(H)** Correlation between CCL3 and CCR5 expression in CRC tissues. **(I)** CCR5-neutralizing antibodies abolish differences in Treg chemotaxis between the siNC and siPin1 groups.

Chemokines play a pivotal role in modulating tumor development within the TME by directing the migration of immune or immunosuppressive cells (21). Tregs are attracted by various CCL chemokines released by both tumor and mesenchymal cells (22). Therefore, we analyzed the relationship between Pin1 and CCL chemokines. The results revealed a significant positive correlation between CCL3 and Pin1 (**Figure 5A**). To validate CCL3 regulation by Pin1, we knocked down Pin1 in SW480 and HT29 cells. qRT-PCR (**Figure 5B**) and ELISA (**Figure 5C**) confirmed reduced CCL3 levels following Pin1 knockdown.

Peripheral blood was procured from healthy volunteers to isolate PBMCs. CD3+ CD4+ CD25+ CD127-/low Tregs were purified from PBMCs by FACS (**Figure 5D**). When co-cultured with Pin1-knockdown CRC cells, the level of Treg markers (CD25+CD127−) were significantly reduced in PBMCs compared to the NC group (**Figures 5E, F**), indicating impaired Treg activation. Chemotaxis assays showed decreased Treg migration toward supernatants from Pin1-knockdown cells (**Figure 5G**). This effect was reversed by adding recombinant CCL3 to the conditioned medium (**Figure 5G**). CCL3 has been reported to influence tumor progression by attracting CCR5+ Tregs within the TME (23). Analysis of TCGA database revealed a robust positive correlation between CCL3 and CCR5 expression levels in CRC patients (**Figure 5H**). CCR5-neutralizing antibody abolished the PIN1 knockdown-induced reduction in Treg migration, which confirms Pin1 regulates Treg chemotaxis via CCL3-CCR5 axis (**Figure 5I**). These results establish the Pin1-CCL3-CCR5 signaling pathway as a key mediator of Treg chemotaxis in the CRC microenvironment.

### 3.6 Pin1 mediated fibroblast activation to inhibit IFN-γ+ CD8+ T cell infiltration

**Figure 6:**
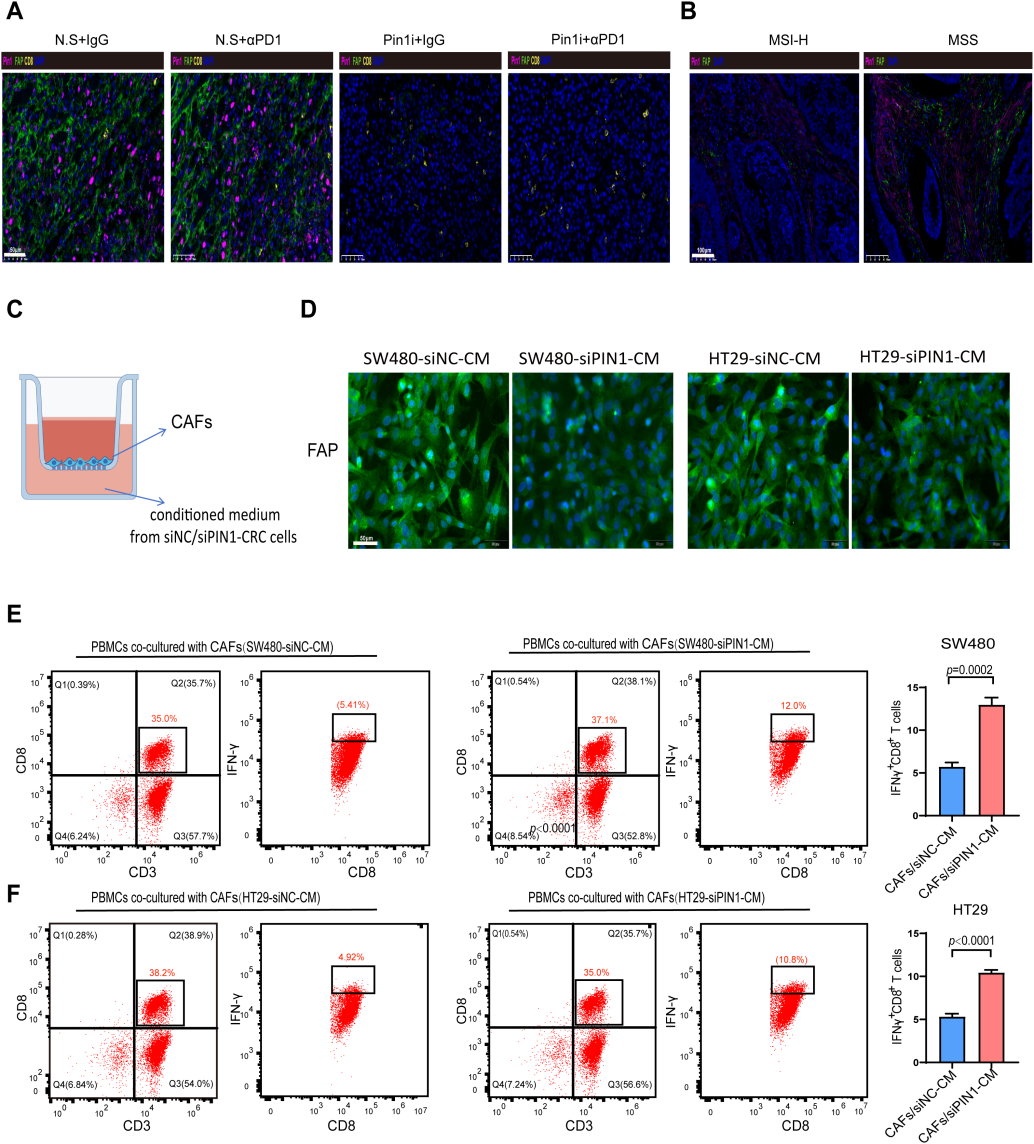
Pin1 mediates fibroblast activation to inhibit IFN-γ+ CD8+ T cell infiltration. **(A)** Representative mIHC staining images of Pin1, FAP, and CD8+T cells in subcutaneous tumors in mice (n=5 per group). **(B)** Representative mIHC staining images of Pin1, FAP, and CD8+T cells in tumor tissues from MSS and MSI-H CRC patients (MSI-H, n=18; MSS, n=24). **(C)** Schematic of transwell culture of CAFs in conditioned medium from siNC- or siPin1-CRC cells. **(D)** IF detection of FAP expression in CAFs after incubation with conditioned media. **(E, F)** Flow cytometry analysis of the percentage of IFN-γ+CD8+ T cells within PBMCs co-cultured with CAFs activated by SW480-siPin1 or HT29-siPin1 cells.

Analysis of the single-cell sequencing data (GSE178341) revealed fibroblasts abundance in the high-Pin1 group compared to the low-Pin1 group in MSS CRC (**Figure 3A**). Cancer-associated fibroblasts (CAFs) are a major component of the tumor stroma, and they can secrete cytokines and chemokines to recruit immunosuppressive cells and inhibit the infiltration and function of effector T cells, thereby shaping the immunosuppressive TME. Immunofluorescence (IF) and IHC analyses demonstrated that treatment with a Pin1 inhibitor led to a reduction in CAF activation in both subcutaneous tumors and liver metastases (**Figure 6A**, **Supplementary Figure S5**). Furthermore, mIHC revealed that Pin1 was expressed higher in the mesenchyme of MSS CRC compared to MSI-H CRC, with concomitant elevation of fibroblast activation protein (FAP) expression (**Figure 6B**). To determine Pin1’s role in CAF activation by CRC cells, we performed indirect co-culture of CAFs with siPin1- or siNC-transfected CRC cells (**Figure 6C**). The findings indicated that FAP expression was significantly reduced in CAFs exposed to conditioned medium from Pin1-knockdown CRC cells compared with controls (**Figure 6D**). When PBMCs were co-cultured with CAFs pre-activated by conditioned medium, flow cytometry revealed increased frequency of IFN-γ+ CD8+ T cells in cultures containing CAFs stimulated by Pin1-knockdown cells (**Figures 6E, F**). These findings demonstrate that Pin1 is a key regulator of CAF activation in MSS CRC. Pin1 inhibition attenuates CAF activation and enhances T cell responses, highlighting its therapeutic potential for modulating the TME and improving immunotherapy efficacy.

### 3.7 Pin1 regulated CCL3 expression to promote Treg cell recruitment and CAF activation through the NF-κB signaling pathway

**Figure 7:**
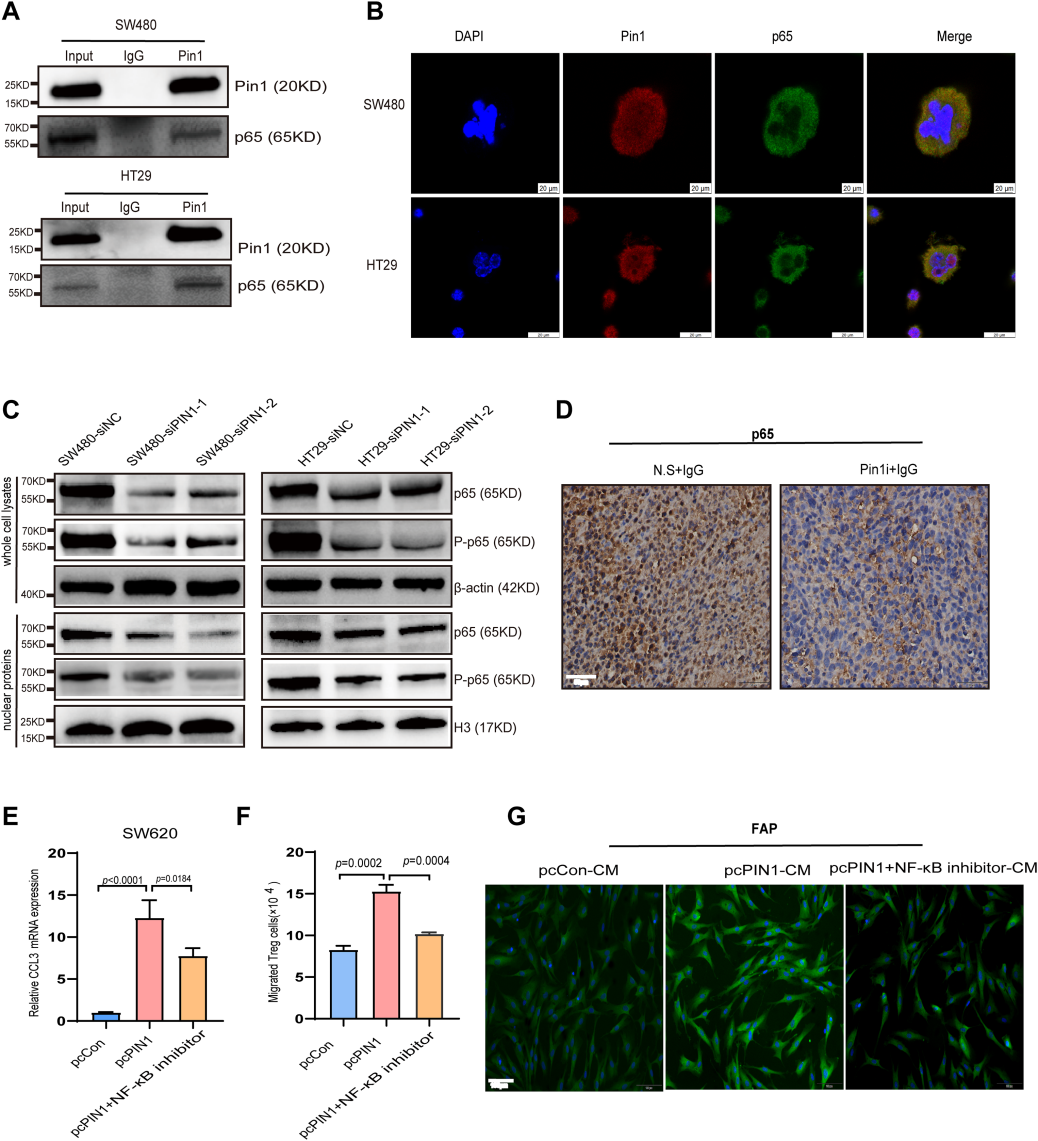
Pin1 regulates CCL3 expression to promote Treg recruitment and CAFs activation through the NF-κB signaling pathway. **(A)** Validation of Pin1 interaction with p65 using CO-IP. **(B)** Co-localization of Pin1 with P65 confirmed using IF. **(C)** The main components of the NF-κB signaling pathway on knockdown of Pin1, as determined using WB. **(D)** Representative images of p65 IHC staining in subcutaneous tumors in mice (N.S. + IgG, Pin1 + IgG) (n=5 per group). **(E)** qRT-PCR assessed CCL3 expression in SW620 cells after Pin1 overexpression with/without NF-κB inhibitor treatment. **(F)** NF-κB inhibitor eliminated Pin1-overexpression-induced differences in Treg chemotaxis. **(G)** NF-κB inhibitor abrogated the activating effect of Pin1 overexpression on CAFs, as analyzed via IF.

PIN1 is reported to activate P65 TF in specific cancers (24, 25). To investigate whether Pin1 interacts with p65 in CRC cells, endogenous co-IP was conducted in SW480 and HT29 cells, confirming their interaction (**Figure 7A**, **Supplementary Figure S2**). Furthermore, Pin1 and p65 were primarily colocalized within SW480 and HT29 cells, as observed by IF analysis (**Figure 7B**). As P65 TF is a core NF-κB component regulating inflammatory factors and chemokines, we examined Pin1’s effect on NF-κB activation. Silencing Pin1 notably decreased P-p65 (Ser276) levels and decreased nuclear p65 and P-p65 levels in SW480 and HT29 cells (**Figure 7C**, **Supplementary Figure S2**). Moreover, the Pin1 inhibitor reduced p65 expression in subcutaneous tumor tissues in mice, as detected by IHC (**Figure 7D**), indicating Pin1-mediated NF-κB activation. When Pin1 was overexpressed in SW620 cells (**Supplementary Figure S6**), we found that inhibition of the NF-κB pathway mitigated the increase in CCL3 induced by Pin1 overexpression (**Figure 7E**). In addition, the NF-κB pathway inhibitor (SC75741, MCE, cat.no. HY-10496) eliminated the differences in Treg chemotaxis and activation of CAFs in co-culture assays (**Figures 7F, G**). Collectively, these results underscore that the pivotal role of Pin1 in regulating the immune response in CRC by activating the NF-κB signaling pathway, which subsequently promotes the recruitment of Treg cells and activation of CAFs through the expression of CCL3.

## 4 Discussion

The management of immunosuppressive TME is a promising strategy for treating various malignancies (26, 27). In this study, we explored differentially expressed genes in MSS and MSI-H patients with CRC. MSI encompasses MSI-H and MSI-L subtypes. Given the overlapping immunotherapy resistance mechanisms between MSS and MSI-L (28), our analysis incorporated MSI-L into the MSS group, focusing on the clinically significant distinction between MSS and MSI-H. Among the identified genes, PIN1 has emerged as a significant candidate because of its substantial correlation with immune cell activity and immune response dynamics. Pin1 is thought to potentially regulate protein function and impact cell cycle regulation and tumor formation (29). In numerous tumor types, Pin1 accelerates tumor development and correlates with unfavorable patient prognosis (30–32). Pin1 inhibition has demonstrated success in sensitizing tumors within an immunosuppressive environment, thereby enhancing their treatability (33). Pin1 serves as the primary regulator within the intricate network of signaling pathways that contribute to cancer drug resistance. Researchers have used small-molecule inhibitors to investigate how inhibiting Pin1 influences drug resistance. For example, Yuan S et al. developed a dual-action, programmable immunoprobiotic delivery system (EcN@Nbs-NP@API-1) that combining Pin1 inhibition and PD-L1 blockade to enhance immunotherapy. This system uses Escherichia coli Nissle 1917 (EcN) to selectively deliver nanoparticles encapsulating the Pin1 inhibitor API-1 to PDAC, which presenting a promising platform to overcome immunotherapy resistance in solid tumors (34). Wu W et al. reviewed that targeting Pin1 as a promising strategy to overcome resistance to cancer therapies. Among many inhibitors reviewed, juglone, epigallocatechin-3-gallate (EGCG), ARTA, and arsenic trioxide (ATO) have exhibited good effects in chemosensitivity and reversal of tumor drug resistance via intervening in cancer-driving pathways, epithelial-mesenchymal transition (EMT) induction and CSCs properties (35). There is no doubt that combination therapies targeting Pin1-related cancer signaling pathways or the creation of novel Pin1-specific inhibitors, will open up new treatment options for both the prevention and management of cancers.

We explored the expression of Pin1 in MSS CRC and its impact on immunotherapy. Our findings indicated a notable overexpression of Pin1 in samples derived from patients with MSS, and a similar pattern was observed in human CRC cell lines. Functionally, silencing Pin1 led to reduced proliferation, increased apoptosis, and diminished migratory ability in SW480 and HT29 cells, suggesting Pin1 may promote the malignant phenotype of MSS CRC cells. To assess translational potential, we explored whether targeting Pin1 could improve immunotherapy response in MSS CRC. According to the manufacturer’s instructions and previous studies, we administered sulfopin (40 mg/kg, intraperitoneal injection, daily) in the animal studies (20, 32). As a highly selective covalent inhibitor targeting Pin1, existing research generally confirms Sulfopin’ s high selectivity: Dubiella C et al. screened an electrophilic fragment library to identify covalent inhibitors targeting Pin1’ s active site Cys113, leading to the development of Sulfopin (a nanomolar Pin1 inhibitor) (36). Two independent chemoproteomics methods validated its high selectivity, and it was shown to achieve potent cellular and *in vivo* target engagement while phenocopying Pin1 genetic knockout, effectively mitigating concerns about potential off-target effects (36). Consistent with our hypothesis, the Pin1 inhibitor + aPD-1 group exhibited the slowest tumor growth and the smallest tumor volume, indicating Pin1 blockade enhances immunotherapy efficacy in MSS models. This aligns with efforts by others to overcome immunotherapy resistance in MSS CRC, such as those by Liu et al., who showed that IL-17A inhibition enhances anti-PD-1 efficacy (37), and Wang H. et al., who identified GBP2 as a promising ICB combination target (8).

Pin1 may impair the efficacy of immunotherapy by modulating the infiltration of immune cells. In Pin1 inhibitor + aPD-1 treated mice, we observed increased CD4+/CD8+ T cells, and decreased Treg recruitment. Single-cell analysis of MSS CRC (GSE178341) revealed higher proportions of fibroblasts, M2 macrophages, and Tregs, but lower CD4+/CD8+ T cells in high-Pin1 samples, indicating an immunosuppressive state. Multiplex IF in human samples corroborated these findings. Collectively, these results suggest Pin1 inhibition reduces MSS CRC tumor growth through enhanced CD4+/CD8+ T cell accumulation and reduced Treg recruitment. We acknowledge that MDSCs and other myeloid subpopulations are well-documented as major drivers of immunosuppression in MSS CRC. They suppress anti-tumor T cell activity via the secretion of arginase-1, inducible nitric oxide synthase (iNOS), and reactive oxygen species (ROS) (38). The lack of comprehensive assessment of these myeloid populations represents a limitation of the current study.

Mechanistically, we propose that Pin1 recruits Tregs via chemokine modulation. We identified a strong positive correlation between Pin1 and CCL3, and Pin1 knockdown significantly inhibited CCL3 secretion, indicating Pin1 regulates CCL3. Chemokine pathways critically govern immune cell recruitment into the TME, influencing tumor progression and therapy response (21). Chemokines recruit immunosuppressive cells (MDSCs, Tregs, M2 macrophages) (39, 40), promote EMT, activate oncogenic pathways (Akt, ERK1/2, NF-κB), and contribute to therapy resistance (22, 41). Zhang et al. linked CCL3/6/8-CCR1 axis to myeloid recruitment and immunosuppression in pancreatic cancer (42), and Liang et al. showed APOE+TAM recruits Tregs via CCL3-CCR5 in iCCA (43). Experimentally, supernatants from Pin1-knockdown cells reduced Treg chemotaxis, while rhCCL3 restored migration, suggesting Pin1 regulates Treg chemotaxis via CCL3. CCR5, a specific CCL3 receptor, is implicated in malignant progression via the CCL3-CCR5 axis (e.g., enhancing migration/invasion in CRC and ESCC through Akt/ERK) (44, 45). TCGA analysis confirmed a robust CCL3-CCR5 correlation in CRC. Using a CCR5-neutralizing antibody, we demonstrated that Pin1 affects Treg chemotaxis via the CCL3-CCR5 pathway, supporting the hypothesis Pin1-CCL3-CCR5 axis regulating Treg chemotaxis in CRC.

Critically, we provide evidence that linking tumor cell-intrinsic Pin1 to stromal reprogramming. Pin1 regulates the immune microenvironment by altering CAF activity. The immunosuppressive TME consists of the ECM, CAFs, various immunosuppressive cells (Tregs, TAMs, TANs, and MDSCs), vasculature system, various inhibitory cytokines, exosomes, and various immune checkpoint molecules (46). CAFs are key immunosuppressive regulators in CRC (47, 48), hindering CD8+ T cell infiltration (48). Single-cell sequencing revealed increased fibroblasts in high-Pin1 MSS CRC. Strikingly, *in vivo* Pin1 inhibitor treatment decreased CAF activation in mouse tumors. Importantly, Pin1 was highly expressed in MSS CRC mesenchyme alongside elevated FAP. Co-culture experiments directly linked tumor cell Pin1 to CAF function: Pin1-knockdown tumor cells reduced FAP expression, and CAFs activated by these cells showed impaired ability to suppress IFN-γ+ CD8+ T cells in PBMC co-cultures. Critically, FAP is not merely a marker but a functional mediator of CAF immunosuppression. Its downregulation directly reflects impaired CAF activation (49). This demonstrates that the expression of Pin1 in tumor cells reprograms CAFs to foster an immunosuppressive TME.

Pin1 interacts with p65, prompting us to investigate whether Pin1 regulates CCL3-CCR5 via activating the NF-κB pathway. Multiple published studies have established P65 TF as a direct transcriptional regulator of CCL3 in similar biological contexts. For example, a key study by Park et al. confirmed NF-κB binding to the CCL3 promoter via ChIP-qPCR in macrophages (50). Similar mechanisms were reported in breast cancer cells (51). Pin1 knockdown inhibited p65 activation in cells and animals. NF-κB inhibition reversed Pin1-overexpression-induced CCL3 upregulation in SW620 cells and attenuated differences in Treg chemotaxis and CAF activation. However, NF-κB inhibition did not fully rescue Pin1 effects, suggesting potential involvement of additional Pin1-regulated pathways. Pin1 facilitates the cancer progression through regulating different substrates at several levels (52). While confirming the essential role of the NF-κB pathway, these findings also highlight the complexity of the signaling network. Collectively, our findings demonstrate that Pin1 promotes the recruitment of Tregs and activates CAFs by upregulating CCL3 via activation of the NF-κB pathway, thereby inhibiting immunotherapy in MSS CRC.

Despite the novel insights from our study, several limitations should be noted. First, while we confirmed Pin1’s role in tumor growth and immunotherapy response, survival analyses of the TCGA cohort showed no significant association between Pin1 expression and overall survival (p = 0.14), likely due to cohort heterogeneity, which limits Pin1’s value as a prognostic marker here. Second, though Pin1 inhibition has translational potential, no Pin1 inhibitors have entered clinical trials, with unresolved challenges like *in vivo* delivery efficiency, long-term safety, and patient-specific responsiveness in CRC (53). Third, while the Pin1 inhibitor Sulfopin has high selectivity, its potential off-target effects in long-term/high-dose use cannot be fully excluded, requiring further validation in larger animal models or early-phase trials. These limitations offer key directions for future research to refine Pin1’s role and advance its translational value in MSS CRC.

## 5 Conclusion

In summary, within the MSS CRC microenvironment, Pin1 drives Treg chemotactic recruitment and CAF activation via the NF-κB-CCL3-CCR5 axis, a mechanism supported by correlative and partial experimental evidence. This remodels the immunosuppressive microenvironment, leading to the progression of MSS CRC and immunotherapy resistance. Given its clinical significance, it may serve as a potential target for MSS CRC immunotherapy.

## Data Availability

The original contributions presented in the study are included in the article/**Supplementary Material**, further inquiries can be directed to the corresponding author/s.
