# Supplementary material for: Targeting Pin1 to overcome immunosuppressive tumor microenvironment in MSS colorectal cancer
Source: Front Immunol. 2025 Oct 31;16:1677029. doi: 10.3389/fimmu.2025.1677029 (PMC12615396; doi:10.3389/fimmu.2025.1677029)
Supplement: Supplementary file 1 [file Table1.docx]

**Supplementary material for**

**Targeting Pin1 to Overcome Immunosuppressive Tumor Microenvironment in pMMR/MSS Colorectal Cancer**

Jian Wang^1, 2#^, Shuxin Tang^3#^, Jinhua Fan^4^, Huihui Xiao^1, 2^, Shihua Wang^5^, Jianqin Xiang^1, 2^, Zhili Liu^6^, Hongying Liu^1, 2^, Zhigang Pei^7^, Dequan Jiang^8^, Leiyuan Shuai^9^, Han Liu^10^, Jian Ye ^11^, Jianli Xu^8^ , Chengyuan Qian^1, 2^, Siqian Cui^2, 12*^, Debing Xiang^1, 2*^, Chunrong Wu^1, 2*^

^1^ Department of Oncology, Chongqing University Jiangjin Hospital, Chongqing, 402260, PR China

^2^ Department of Oncology, Jiangjin Central Hospital of Chongqing, Chongqing 402260, PR China

^3^ Department of Neonatology, The Sixth Affiliated Hospital, Sun Yat-sen University, Guangzhou, Guangdong 510655, China

^4^ Central Laboratory, Chongqing University Fuling Hospital, Chongqing University, Fuling, Chongqing 408000, China.

^5^ The First Medicine College, Chongqing Medical University, Chongqing, 400014, PR China

^6^ Department of Cardiothoracic Surgery, Southwest Hospital Third Military Medical University, Chongqing, 400038, PR China

^7^ Department of Pathology, Chongqing University Jiangjin Hospital, Chongqing, 402260, PR China

^8^ Department of Gastrointestinal Surgery, Chongqing University Jiangjin Hospital, Chongqing, 402260, PR China

^9^ Department of Anus and intestine surgery, Chongqing University Jiangjin Hospital, Chongqing, 402260, PR China

^10^ Department of Ophthalmology, Chongqing University Jiangjin Hospital, Chongqing, 402260, PR China

^11^ Department of Anesthesiology, Chongqing University Jiangjin Hospital, Chongqing, 402260, PR China

^12^ College of Chemistry and Chemical Engineering, Chongqing University, Chongqing, 400044, PR China.

Corresponding authors:

Dr. Siqian Cui (MD); E-mail: cuisq742278@163.com

Prof. Dr Debing Xiang (MD); E-mail: xdb86@cqu.edu.cn

Prof. Dr Chunrong Wu (MD); E-mail: [cqwcr6688@cqu.edu.cn](mailto:cqwcr6688@cqu.edu.cn)

**This PDF file includes:**

**Table S1.** Primer pairs used for qRT-PCR.

**Table S2.** Clinicopathological features of patients with CRC.

**Table S3.** Antibodies used for western blotting, immunohistochemistry, multiple immunohistochemistry, immunoﬂuorescence, Immunoprecipitation and flow cytometric assay.

**Figure S1.** Flow cytometry gating strategies.

**Figure S2.** The original uncropped image of the blots used in the figure of our manuscript.

**Figure S3.** Pin1 expression in mouse CRC cell lines.

**Figure S4.** Cellular composition of TME in CRC from GSE178341.

**Figure S5.** Expression of FAP in hepatic metastasis mice tissue.

**Figure S6.**Efficacy of Pin1 overexpression vectors.

**Table S1. Primer pairs used for qRT-PCR.**

| **name of primer** | **sequence (5'-3')** |
| --- | --- |
| H-PIN1-qPCR-F | GCAACAGCAGCAGTGGTG |
| H-PIN1-qPCR-R | CAAAGTCCTCCTCTCCCGA |
| H-TUFM-qPCR-F | CCAGACCCGAGAGCACTTA |
| H-TUFM-qPCR-R | GAGCAGAGCCTACGATGAC |
| H-β-actin-qPCR-F | ACCCCGTGCTGCACCGAG |
| H-β-actin-qPCR-R | TCCCGGCCAGCCAGGTCCA |

**Table S2. Clinicopathological features of patients with CRC.**

| **Clinicopathological Feature** | **n= 40** | **MSS**  **n (%)** | **MSI-H**  **n (%)** | **p-value^2^** |
| --- | --- | --- | --- | --- |
| **Gender** |  |  |  | 0.44 |
| Male | 17 | 8 (33.33) | 9 (50.00) |  |
| Famale | 25 | 14 (66.67) | 9 (50.00) |  |
| **Age (yrs) (mean ± SD)** |  | 62.76 ± 10.01 | 63.22 ±11.05 | 0.80 |
| **Tumor Location** |  |  |  | 0.16 |
| Left hemicolon | 10 | 3 (12.50) | 7 (38.89) |  |
| Right hemicolon | 11 | 7 (29.17) | 4 (22.22) |  |
| rectum | 21 | 14 (58.33) | 7 (38.89) |  |
| **Differentiation** |  |  |  | 0.13 |
| well | 9 | 3 (12.50) | 6 (33.33) |  |
| moderate | 26 | 18 (75.00) | 8 (44.44) |  |
| poor | 7 | 3 (12.50) | 4 (22.22) |  |
| **Lymph Node Metastasis** |  |  |  | 0.26 |
| - | 17 | 12 (50.00) | 5 (27.78) |  |
| + | 25 | 12 (50.00) | 13 (72.22) |  |
| **Distant Metastasis** |  |  |  | 0.96 |
| - | 29 | 16 (66.67) | 13 (72.22) |  |
| + | 13 | 8 (33.33) | 5 (27.78) |  |
| **TNM** |  |  |  | >0.99 |
| I-II | 23 | 13 (54.17) | 12 (55.56) |  |
| III-IV | 19 | 11(45.83) | 6 (44.44) |  |

**Table S3. Antibodies used for western blotting, immunohistochemistry, multiple immunohistochemistry, immunoﬂuorescence, Immunoprecipitation and flow cytometric assay**

| **Antibody** | **Source** | **Product No.** | **maker** | **Experiment** |
| --- | --- | --- | --- | --- |
| Pin1 | mouse monoclonal antibody | sc-46660 | Santa cruz | Western blotting（1:200） Immunohistochemistry（1:100） Multiple immunohistochemistry（1:100） Immunoﬂuorescence（1:100） Immunoprecipitation（1ug/200ug protein） |
| FAP | Rabbit polyclonal antibody | ab53066 | abcam | Multiple immunohistochemistry (1:100） Immunoﬂuorescence （1:100） |
| NF-KB P65 | Rabbit polyclonal antibody | 10745-1-AP | Proteitech | Western blotting （1:200） |
| p-NFκB p65 (Ser276) | Rabbit Polyclone antibody | sc-101749 | Santa cruz | Western blotting （1:200） |
| CD4 | mouse monoclonal antibody | sc-19641 | Santa cruz | Multiple immunohistochemistry（1:100） |
| CD8 | mouse monoclonal antibody | sc-1177 | Santa cruz | Multiple immunohistochemistry（1:100） |
| FOXP3 | mouse monoclonal antibody | ab20034 | abcam | Multiple immunohistochemistry（1:100） |
| β-actin | mouse monoclonal antibody | 66009-1-Ig | Proteintech | Western blotting （1:10000） |
| CD45 Monoclonal Antibody (30-F11), APC-eFluor™ 780 | Rat monoclonal antibody | 47-0451-82 | eBioscience™ | Flow cytometric （1:100） |
| CD3e Monoclonal Antibody (145-2C11), FITC | Armenian hamster monoclonal antibody | 11-0031-81 | eBioscience™ | Flow cytometric （1:100） |
| CD4 Monoclonal Antibody (GK1.5), PE | Rat monoclonal antibody | 12-0041-81 | eBioscience™ | Flow cytometric （1:100） |
| CD8a Monoclonal Antibody (53-6.7), APC | Rat monoclonal antibody | 17-0081-82 | eBioscience™ | Flow cytometric （1:100） |
| F4/80 Monoclonal Antibody (BM8), PE | Rat monoclonal antibody | 12-4801-80 | eBioscience™ | Flow cytometric （1:100） |
| CD206 (MMR) Monoclonal Antibody (MR6F3), APC | Rat monoclonal antibody | 17-2061-80 | eBioscience™ | Flow cytometric （1:100） |
| CD11b Monoclonal Antibody (M1/70), Super Bright™ 600 | Rat monoclonal antibody | 63-0112-82 | eBioscience™ | Flow cytometric （1:100） |
| Ly-6G Monoclonal Antibody (1A8-Ly6g), PE | Rat monoclonal antibody | 12-9668-82 | eBioscience™ | Flow cytometric（1:100） |
| Ly-6C Monoclonal Antibody (HK1.4), APC | Rat monoclonal antibody | 17-5932-82 | eBioscience™ | Flow cytometric （1:100） |
| CD3 Monoclonal Antibody (SK7), APC-eFluor™ 780 | mouse monoclonal antibody | 47-0036-42 | eBioscience™ | Flow cytometric（1:100） |
| CD4 Monoclonal Antibody (RPA-T4), FITC | mouse monoclonal antibody | 11-0049-42 | eBioscience™ | Flow cytometric（1:100） |
| CD25 Monoclonal Antibody (BC96), Brilliant Violet™ 421 | mouse monoclonal antibody | 404-0259-42 | eBioscience™ | Flow cytometric（1:100） |
| CD127 Monoclonal Antibody (eBioRDR5), PE | mouse monoclonal antibody | 12-1278-42 | eBioscience™ | Flow cytometric （1:100） |
| CD8a Monoclonal Antibody (RPA-T8), perCP-Cyanine5.5 | mouse monoclonal antibody | 45-0088-42 | eBioscience™ | Flow cytometric （1:100） |
| IFN gamma Monoclonal Antibody (4S.B3), APC | mouse monoclonal antibody | 17-7319-82 | eBioscience™ | Flow cytometric（1:100） |

**Figure S1.**

**
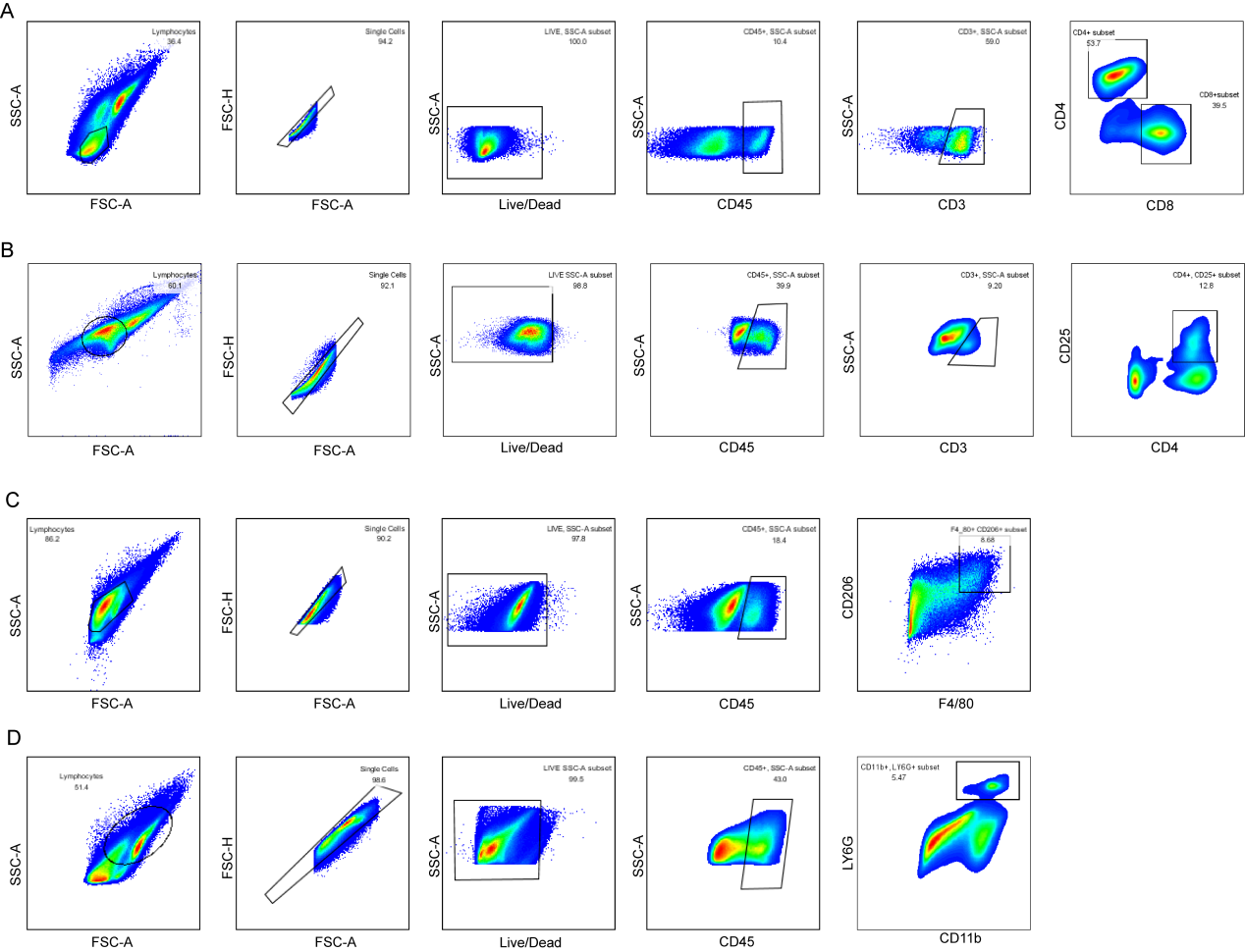
**

**Figure S1. Flow cytometry gating strategi**es. (A) Gating strategy followed to determine T-cell subpopulations. A representative dot plot is shown. The gating strategy was the follow: Lymphocytes, Singlets, Live cells, CD45+ cells, CD3+ T cells, CD4+ and CD8+ T cells. (B) Gating strategy followed to determine Treg subpopulations. The gating strategy was as follows: Lymphocytes, Singlets, Live cells, CD45+ cells, CD3+ T cells, CD4+ and CD25+ Treg cells. (C) Gating strategy for M2 macrophages. A representative dot plot is shown. The gating strategy was as follows: Myeloid, Singlets, Live cells, CD45+ cells, F4/80+ and CD206+ cells. (D) Gating strategy for neutrophils. The gating strategy was shown: Granulocytes, Singlets, Live cells, CD45+ cells, CD11b+ and Ly6G+ cells.

**Figure S2.**

**
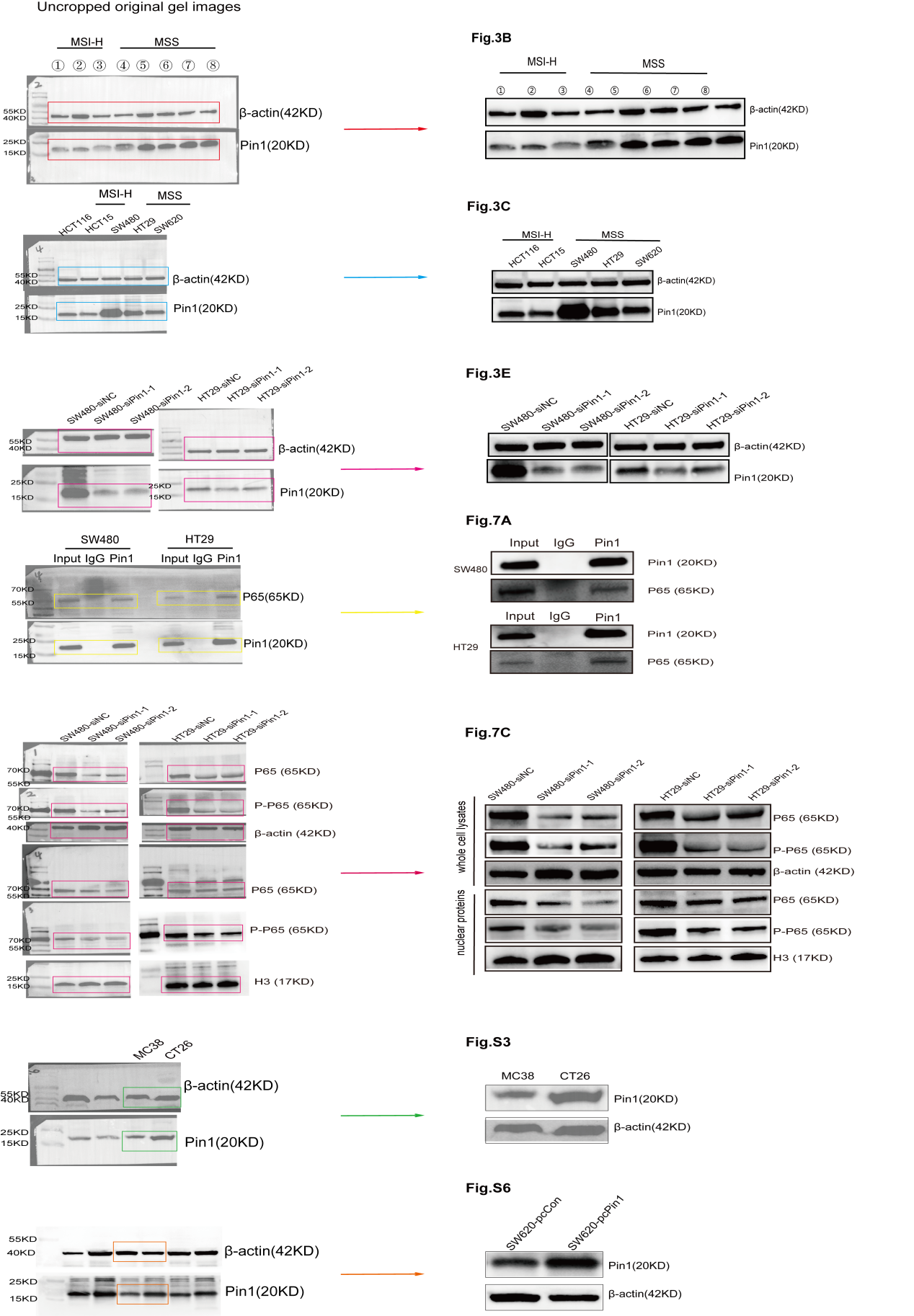
**

**Figure S2.**The original uncropped image of the blots used in the figure of our manuscript.

**Figure S3.**

| **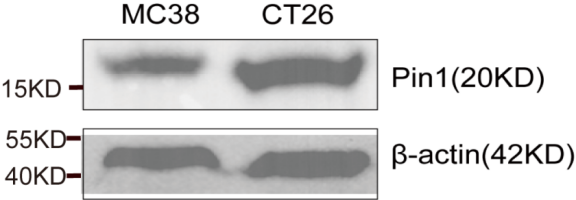** |
| --- |
| **Figure S3. Pin1 expression in mouse CRC cell lines.** Pin1 protein expression level in mouse MSS CRC cell (CT26) and MSI CRC cell (MC38). |

**Figure S4.**

| 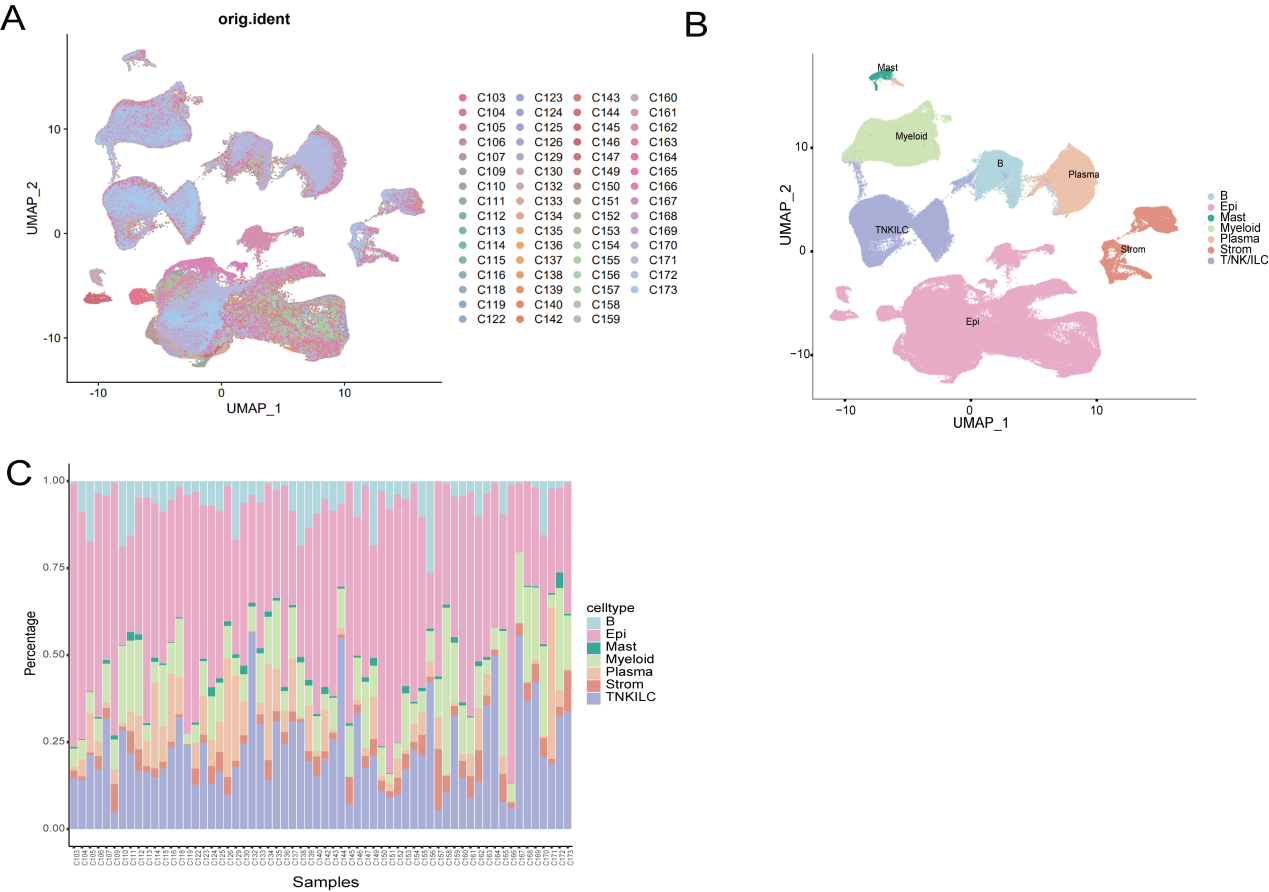 |
| --- |
| **Figure S4. Cellular composition of TME in CRC from GSE178341.** (A) UMAP plot showing the correction of the variation emanating from the batches of individual sample sequencing (B) UMAP visualization of major cell types in CRC. (B)Relative proportions of cell types in each sample. |

**Figure S5.**

**
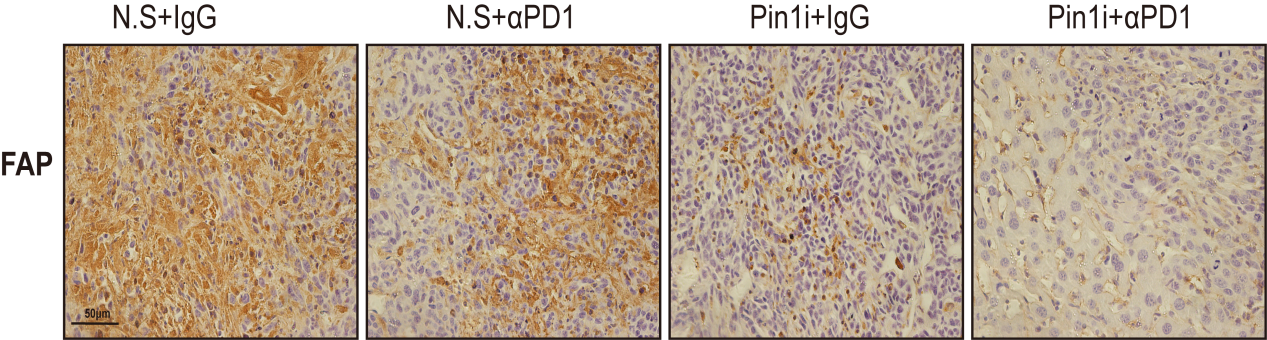
**

**Figure S5.** **Expression of FAP in** **hepatic metastasis mice tissue.** Representative IHC staining images of FAP in hepatic metastasis mice tissue (n=5 per group).

**Figure S6.**

| 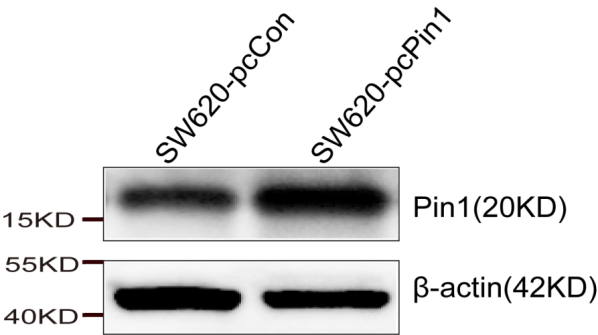 |
| --- |
| **Figure S6. Efficacy of Pin1 overexpression vectors.** Pin1 protein expression level in SW620 cells transfected with pcPin1, as examined using western blotting. Cells transfected with pcCon was used as controls. |
